# Cardiolipin Synthesis in Brown and Beige Fat Mitochondria Is Essential for Systemic Energy Homeostasis

**DOI:** 10.1016/j.cmet.2018.05.003

**Published:** 2018-07-03

**Authors:** Elahu G. Sustarsic, Tao Ma, Matthew D. Lynes, Michael Larsen, Iuliia Karavaeva, Jesper F. Havelund, Carsten H. Nielsen, Mark P. Jedrychowski, Marta Moreno-Torres, Morten Lundh, Kaja Plucinska, Naja Z. Jespersen, Trisha J. Grevengoed, Barbara Kramar, Julia Peics, Jakob B. Hansen, Farnaz Shamsi, Isabel Forss, Ditte Neess, Susanne Keipert, Jianing Wang, Katharina Stohlmann, Ivan Brandslund, Cramer Christensen, Marit E. Jørgensen, Allan Linneberg, Oluf Pedersen, Michael A. Kiebish, Klaus Qvortrup, Xianlin Han, Bente Klarlund Pedersen, Martin Jastroch, Susanne Mandrup, Andreas Kjær, Steven P. Gygi, Torben Hansen, Matthew P. Gillum, Niels Grarup, Brice Emanuelli, Søren Nielsen, Camilla Scheele, Yu-Hua Tseng, Nils J. Færgeman, Zachary Gerhart-Hines

**Affiliations:** 1 Novo Nordisk Foundation Center for Basic Metabolic Research, University of Copenhagen, Copenhagen 2200, Denmark; 2 Department of Biomedical Sciences, University of Copenhagen, Copenhagen 2200, Denmark; 3 Section on Integrative Physiology and Metabolism, Joslin Diabetes Center, Harvard Medical School, Boston, MA 02215, USA; 4 Department of Biochemistry and Molecular Biology, University of Southern Denmark, Odense 5230, Denmark; 5 Department of Clinical Physiology, Nuclear Medicine & PET and Cluster for Molecular Imaging, Rigshospitalet, Copenhagen 2200, Denmark; 6 Department of Cell Biology, Harvard Medical School, Boston, MA 02115, USA; 7 Centre of Inflammation and Metabolism and Centre for Physical Activity Research, Rigshospitalet, University Hospital of Copenhagen, Copenhagen 2200, Denmark; 8 Helmholtz Diabetes Center and German Diabetes Center (DZD), Helmholtz Zentrum München, Neuherberg 85764, Germany; 9 Barshop Institute for Longevity and Aging Studies, University of Texas Health Science Center at San Antonio, San Antonio, TX 78229, USA; 10 Lillebaelt Hospital, Vejle 7100, Denmark; 11 Institute of Regional Health Research, University of Southern Denmark, Odense 5230, Denmark; 12 Steno Diabetes Center, Gentofte 2820, Denmark; 13 National Institute of Public Health, Southern Denmark University, Copenhagen 1353, Denmark; 14 Research Center for Prevention and Health, Glostrup 2600, Denmark; 15 Center for Clinical Research and Prevention, Bispebjerg and Frederiksberg Hospital, Copenhagen, Denmark; 16 Department of Clinical Medicine, University of Copenhagen, Copenhagen 2200, Denmark; 17 BERG Health, Framingham, MA 01701, USA

**Keywords:** cardiolipin, CRLS1, phospholipids, lipid metabolism, mitochondria, brown adipose, beige adipose, thermogenesis, insulin resistance, CHOP-10

## Abstract

Activation of energy expenditure in thermogenic fat is a promising strategy to improve metabolic health, yet the dynamic processes that evoke this response are poorly understood. Here we show that synthesis of the mitochondrial phospholipid cardiolipin is indispensable for stimulating and sustaining thermogenic fat function. Cardiolipin biosynthesis is robustly induced in brown and beige adipose upon cold exposure. Mimicking this response through overexpression of cardiolipin synthase (*Crls1*) enhances energy consumption in mouse and human adipocytes. *Crls1* deficiency in thermogenic adipocytes diminishes inducible mitochondrial uncoupling and elicits a nuclear transcriptional response through endoplasmic reticulum stress-mediated retrograde communication. Cardiolipin depletion in brown and beige fat abolishes adipose thermogenesis and glucose uptake, which renders animals insulin resistant. We further identify a rare human *CRLS1* variant associated with insulin resistance and show that adipose *CRLS1* levels positively correlate with insulin sensitivity. Thus, adipose cardiolipin has a powerful impact on organismal energy homeostasis through thermogenic fat bioenergetics.

## Highlights

• Cardiolipin (CL) is a key effector of brown and beige fat thermogenic programs
• CL mediates mitochondria-to-nucleus crosstalk through the ER stress factor CHOP-10
• Disruption of brown and beige fat mitochondria causes insulin resistance
• CL synthesis in adipose tissue is linked to insulin sensitivity in humans

## Graphical Abstract

**Figure:**
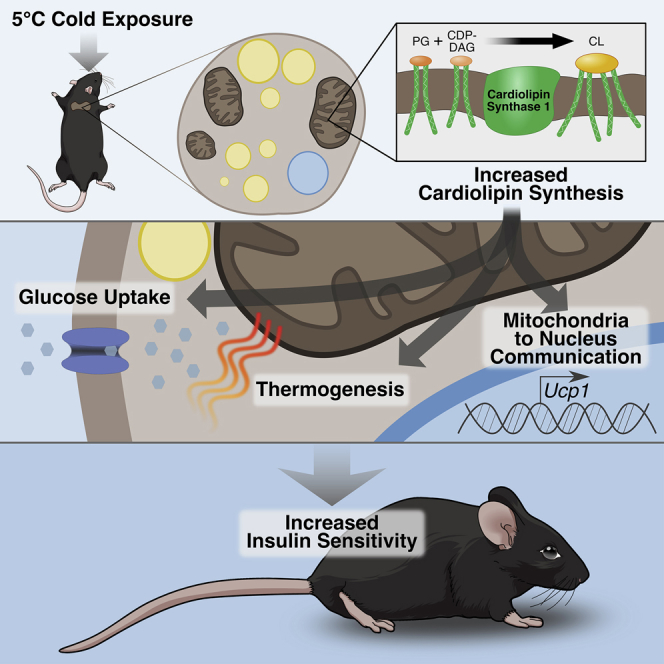

## Teaser

Sustarsic et al. reveal that synthesis of the mitochondrial phospholipid cardiolipin is a hallmark of brown and beige fat activation by cold temperature. This single lipid species in thermogenic fat not only shapes adipose mitochondrial bioenergetics but also exerts profound control over whole-body insulin sensitivity and metabolic flexibility.

## Introduction

Brown adipose tissue (BAT) is a specialized organ that converts carbohydrate and lipid substrates to thermal energy to defend body temperature in response to cold environments (Cannon and Nedergaard, 2004). This core function of BAT depends on robust electron transport activity that is fueled by a capacity for glucose and lipid consumption proportionally greater than any other tissue (Bartelt et al., 2011, Labbe et al., 2015). Another type of cell, known as a “beige” or “brite” adipocyte, can be recruited to become thermogenically active through a process called “browning” (Peirce et al., 2014, Petrovic et al., 2010, Shabalina et al., 2013, Wu et al., 2012). In mice, these cells are located primarily in subcutaneous white adipose tissue (scWAT) and possess thermogenic capacity through both UCP1-dependent (Shabalina et al., 2013) and -independent means (Kazak et al., 2015). In rodents, both brown and beige adipocytes (also known as thermogenic adipocytes) are protective against metabolic disease (Cohen et al., 2014, Feldmann et al., 2009). The presence of thermogenic adipocytes in adult humans (Cypess et al., 2009, van Marken Lichtenbelt et al., 2009, Müller et al., 2016, Saito et al., 2009, Virtanen et al., 2009) has opened up the possibility to exploit these fat cells to treat obesity and diabetes (Betz and Enerbäck, 2015, Harms and Seale, 2013, Kusminski et al., 2016, Lee et al., 2013, Sidossis and Kajimura, 2015).

Exposure to cold temperature is the most robust way to elicit the adipose tissue thermogenic response. Previous analyses of single bouts of cold exposure have yielded valuable insights into cold-induced transcriptional programming (Hao et al., 2015, Marcher et al., 2015), lipid composition (Marcher et al., 2015), UCP1-independent thermogenic mechanisms (Kazak et al., 2015), and comparisons between brown and white adipose responses (Rosell et al., 2014, Shore et al., 2013). However, the ability of adipose tissue to expend energy is a dynamic process that continues to increase with prolonged cold exposure, only reaching maximal capacity after several weeks (Cannon and Nedergaard, 2004). This temporal progression is demonstrated by a brown fat mitochondrial proteomics study that revealed differential changes in respiratory chain complexes between two time points of cold exposure (Forner et al., 2009).

Here we set out to temporally map the global BAT proteome throughout cold adaptation in order to identify key regulators of thermogenesis. We found that lipid metabolism processes were the most dynamic and robustly induced pathways in response to cold. Therefore, we surveyed the cold-regulated lipid landscapes of brown and beige adipose using targeted lipidomics. We found that cardiolipins (CLs) were the lipids most significantly induced by cold in both thermogenic fat depots. CLs are unique phospholipids that are synthesized and predominantly located in the mitochondrial inner membrane. CLs are ascribed numerous roles in mitochondrial biology, including respiratory chain supercomplex formation, cytochrome *c* sequestration, OPA1-mediated fusion, and mitochondrial carrier activation (Houtkooper and Vaz, 2008, Paradies et al., 2014, Shi, 2010). Originally discovered in bovine heart, these lipids have been almost exclusively studied in cardiac and skeletal muscle, brain, and liver (Houtkooper and Vaz, 2008, Shi, 2010). Earlier studies have observed increases in BAT CLs with cold exposure (Ogawa et al., 1987, Ricquier et al., 1978); however, the physiological role of CL in thermogenic fat remains unknown. Here, we demonstrate that the mitochondrial lipid CL shapes brown and beige fat bioenergetics to profoundly affect organismal metabolic fitness and glucose homeostasis.

## Results

### Lipid Metabolism Pathways Dominate the Thermogenic Fat Proteome during Cold Adaptation

**Figure 1:**
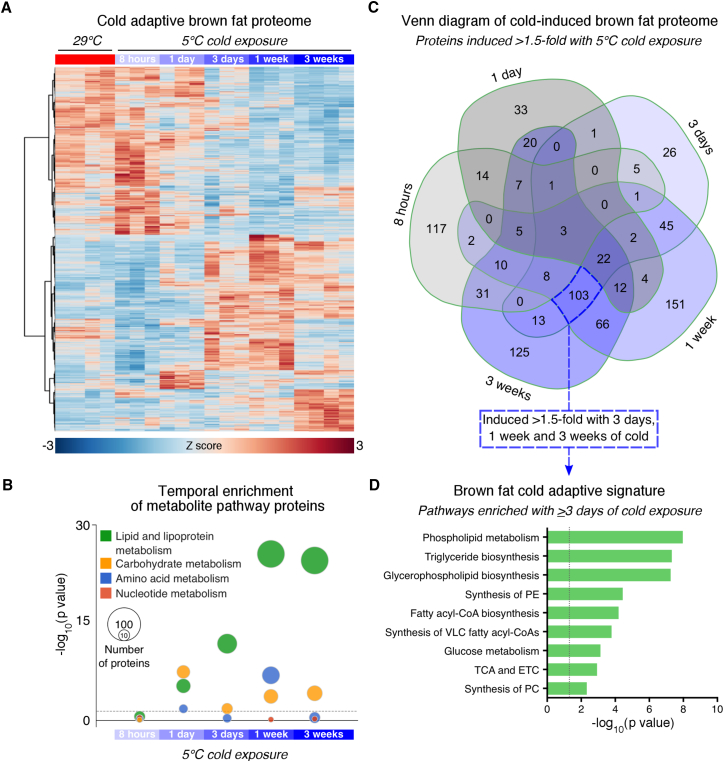
Lipid Metabolism Pathways Are the Key Signature of the Brown Fat Cold Adaptive Proteome (A) Heatmap of *Z* score transformed expression of 1,400 significantly changed proteins in BAT during cold adaptation (n = 4 for 29°C and 5°C 3 weeks, n = 3 for other groups; ANOVA adjusted p value <0.05). (B) Temporal pattern of amino acid, nucleotide, carbohydrate, and lipid metabolism Reactome pathways during cold adaptation. The size of the bubbles indicates the number of pathway proteins upregulated by cold (t test and fold change >1.5). (C) Venn diagram of proteins induced by cold >1.5-fold. (D) Pathway enrichment of common proteins between 3 days, 1 week, and 3 weeks cold that were induced >1.5-fold. Dashed lines indicate adjusted p value <0.05. ETC, electron transport chain; PC, phosphatidylcholine; PE, phosphatidylethanolamine; TCA, tricarboxylic acid. See also Figure S1.

We first set out to obtain an unbiased, temporal view of the cold adaptive protein landscape in thermogenic adipose tissue. We performed semiquantitative, tandem mass tag-mass spectrometry on interscapular BAT (iBAT) from mice housed at thermoneutrality (i.e., lowest thermogenesis) or subjected to 5°C cold challenges ranging from acute (i.e., 8 hr) to full cold adaptation (i.e., 3 weeks). A total of 5,552 detected proteins were common between the two multiplexed datasets. Of these, 1,400 proteins were significantly changed during cold adaptation (Figures 1A and S1A; Data S1). Known markers of BAT activation were highly induced in cold exposed mice (Figure S1B).

To identify time-dependent changes in metabolite pathways, we examined the temporal enrichment of proteins involved in amino acid, nucleotide, carbohydrate, and lipid metabolism (Figure 1B). Both carbohydrate and lipid metabolism pathways were notably enriched after 1 day of cold and sustained throughout cold adaptation. However, the enrichment of lipid metabolism proteins far eclipsed that of proteins involved in all other metabolite pathways from 3 days to 3 weeks of cold exposure (Figure 1B). This shift marked a distinct transition in the cold adaptive proteome. To uncover the most critical processes contributing to this transition, we focused our analysis on proteins significantly upregulated by 3 days, 1 week, and 3 weeks of cold (Figure 1C, dashed blue box). Strikingly, this sub-group represented the largest common overlap between any time points, suggesting a strong core signature. Consistent with our earlier findings on metabolite pathways (Figure 1B), this group of 103 cold-induced proteins was the most enriched for lipid metabolism processes, with phospholipid metabolism, triglyceride biosynthesis, and glycerophospholipid biosynthesis as the top three pathways represented (Figure 1D). Together, these data indicate that the iBAT proteome undergoes a major transition between acute and chronic cold exposure that is dominated by lipid metabolism.

### Environmental Activation of Brown and Beige Fat Robustly Induces Cardiolipin Synthesis

**Figure 2:**
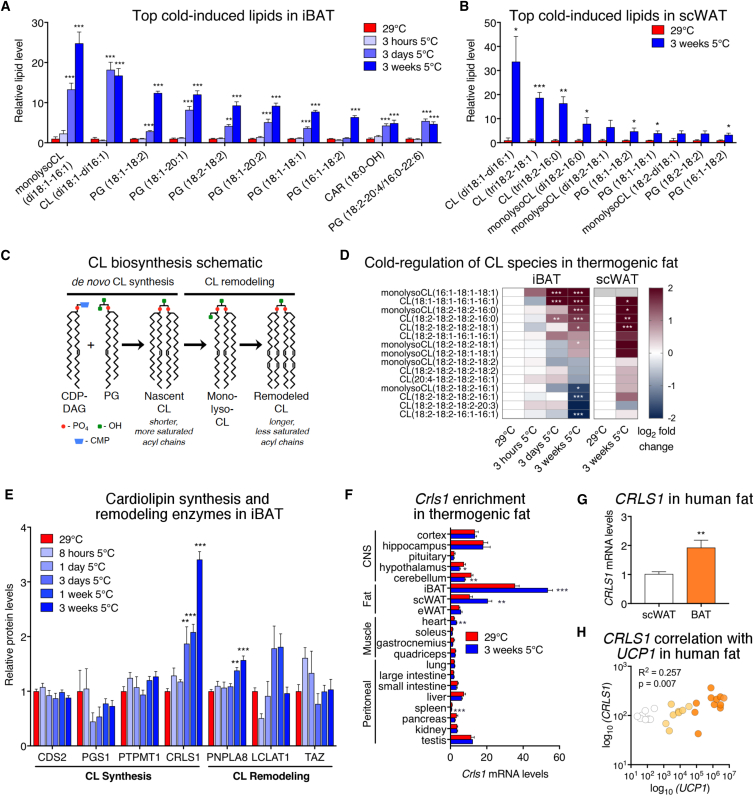
Cardiolipins Are Robustly Increased in Activated Brown and Beige Fat (A) Ten most cold-induced lipids from lipidomic analysis of iBAT from mice housed at thermoneutrality or exposed to 5°C cold for 3 hr, 3 days, or 3 weeks (fold change relative to thermoneutral, 29°C = 1; n = 4 per group, one-way ANOVA). (B) Top ten cold-induced lipids from lipidomic analysis of scWAT from mice housed at thermoneutrality or exposed to 5°C cold for 3 weeks (fold change of absolute quantities normalized to protein content, 29°C = 1; n = 4 per group, t test). CL, cardiolipin; PG, phosphatidylglycerol; CAR, acyl carnitine. (C) Schematic of cardiolipin synthesis and remodeling; CDP-DAG, cytidine diphosphate diacylglycerol; CMP, cytidine monophosphate. (D) Heatmaps of log_2_ (fold change cold treated/thermoneutrality) for CL species in iBAT and scWAT (n = 4 per group, one-way ANOVAs for iBAT and t tests for scWAT; gray, not detected). (E) Relative iBAT protein levels of cardiolipin *de novo* synthesis and remodeling pathway enzymes from proteomic analysis presented in Figure 1A (n = 4 for 29°C and 5°C 3 weeks, n = 3 for other groups; one-way ANOVA). (F) Tissue distribution of *Crls1* from mice housed at thermoneutrality or cold exposed for 3 weeks (n = 6 per group; t test). (G and H) (G) *CRLS1* levels in human subcutaneous fat (n = 8) and brown fat with high *UCP1* mRNA expression (n = 11, *UCP1* CT values between 19 and 27; t test) and (H) correlation with *UCP1* in scWAT (n = 8; white), low-*UCP1* BAT (n = 8, *UCP1* CT values between 27 and 37; yellow), and high-*UCP1* BAT (n = 11; orange; Pearson R^2^ and p value shown). Data are presented as means ±SEM. ^∗^p < 0.05; ^∗∗^p < 0.01; ^∗∗∗^p < 0.001. See also Figure S2.

The profound impact of cold exposure on iBAT lipid-metabolizing enzymes (Figures 1B and 1D) prompted us to interrogate the brown fat lipidome. Using targeted, quantitative mass spectrometry, iBAT lipidomic profiles were obtained from mice housed at thermoneutrality or subjected to a 5°C cold challenge for 3 hr, 3 days, or 3 weeks. Of the 287 lipids surveyed, 250 were significantly altered in iBAT by cold exposure (Figure S2A; Data S2). The most cold-induced lipid species in brown fat were members of the cardiolipin (CL) phospholipid family (Figure 2A). Our findings place previously observed cold induction of cardiolipins (Ogawa et al., 1987, Ricquier et al., 1978) in a more global context of the brown fat lipidome. We additionally obtained lipidomic profiles from scWAT depots at thermoneutrality and after 3 weeks of cold to pinpoint critical lipids conserved between brown and beige depots. Thirty-two lipid species were changed in scWAT following cold adaptation (Figure S2B; Data S2) and, as in iBAT, we found the most cold-induced lipid species in beige fat were also CLs (Figure 2B).

CLs are synthesized in the mitochondrial inner membrane by coupling cytidine diphosphate-diacylglycerol (CDP-DAG) with phosphatidylglycerol (PG) (Figure 2C). Notably, PGs were also among the highest induced lipids in thermogenic fat (Figures 2A, 2B, S2C, and S2D). Newly synthesized CL is characterized by shorter, more saturated acyl chains, which can be remodeled by phospholipases, and acyltransferases through monolysocardiolipin (monolysoCL) intermediates to generate a diverse pool of CLs. This diversity is thought to enable CLs to influence a wide array of mitochondrial functions (Houtkooper and Vaz, 2008, Paradies et al., 2014, Shi, 2010). Among the 15 targeted CL and monolysoCL species measured, we found six were significantly upregulated by cold in iBAT and four in scWAT (Figures 2D and S2E). The most cold-induced CL species in brown and beige adipose were largely nascent or within the early stages of remodeling. Together, these data show that increased CL synthesis is a conserved lipid signature of activated thermogenic brown and beige fat.

### Cardiolipin Synthase 1 Enhances Inducible Mitochondrial Uncoupling in Thermogenic Adipocytes

To identify the enzyme or enzymes responsible for the striking elevation of CLs in activated brown and beige fat, we examined the cold regulation of CL-metabolizing enzymes in iBAT (Figure 2E). CL synthase 1 (CRLS1) was the most robustly induced CL enzyme throughout cold adaptation. Notably, the pattern of CRLS1 induction closely paralleled that of iBAT CL levels (Figure 2A), both of which were significantly increased beginning at 3 days of cold exposure. An examination of gene expression across 20 mouse tissues revealed that *Crls1* was highest enriched in iBAT and was further increased by cold exposure in both iBAT and scWAT (Figure 2F). The Genotype-Tissue Expression (GTEx) database showed that *CRLS1* is also highest expressed in human adipose. Consistent with our mouse data, *CRLS1* expression was more abundant in human supraclavicular brown fat versus subcutaneous fat and correlated with *UCP1* levels (Figures 2G and 2H). Taken together, these data support a role for CRLS1 and CL biosynthesis in mouse and human brown fat.

**Figure 3:**
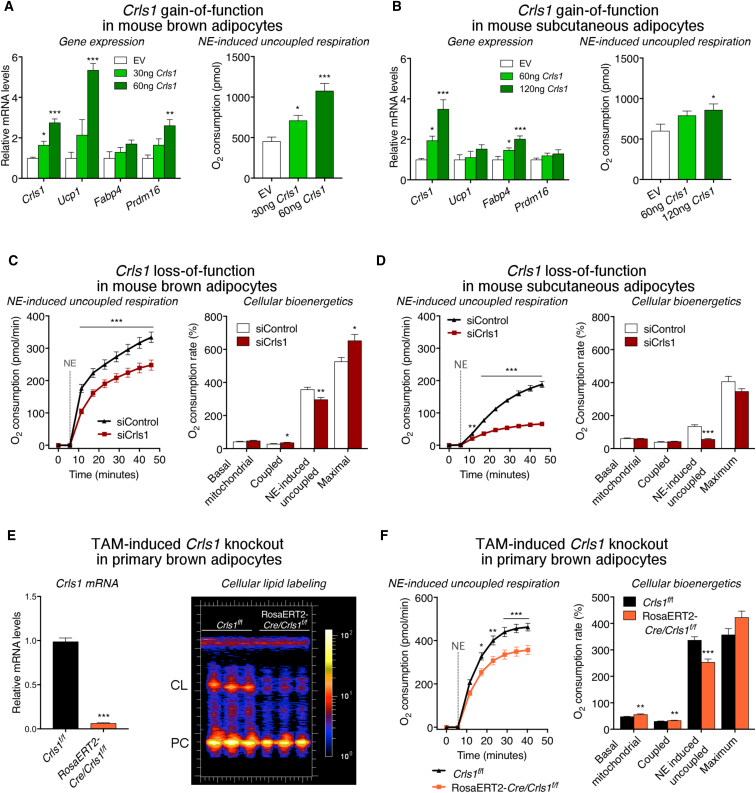
CRLS1 Enhances Inducible Uncoupling in Thermogenic Fat Cells (A–D) Gene expression and NE-induced respiration from primary brown (A) and subcutaneous (B) adipocytes electroporated with either empty pcDNA vector or vector expressing *Crls1* (one-way ANOVA). Oxygen consumption profiles from control and *Crls1* siRNA-treated (C) primary brown and (D) subcutaneous adipocytes following addition of oligomycin and NE stimulation (repeated measures two-way ANOVA). Quantified levels of basal, ATP synthesis-coupled, NE-induced uncoupled, and maximal respiration are provided to the right (t tests). (E and F) (E) *Crls1* mRNA levels and cardiolipin (CL) labeling for 6 hr and (F) oxygen consumption profiles of primary brown adipocytes from control *Crls1*^*f/f*^ and Rosa26ERT2-*Cre*/*Crls1*^*f/f*^ mice treated with tamoxifen (TAM). Data are presented as means ± SEM. ^∗^p < 0.05; ^∗∗^p < 0.01; ^∗∗∗^p < 0.001. See also Figure S3.

To assess the functional consequences of *Crls1* cold induction, we transiently overexpressed *Crls1* in primary mouse brown and subcutaneous adipocytes. Ectopic *Crls1* expression in both cell types significantly enhanced norepinephrine (NE)-induced uncoupled respiration, a hallmark of thermogenic metabolism (Cannon and Nedergaard, 2004) (Figures 3A and 3B). *Crls1* overexpression in brown adipocytes also led to increased *Ucp1* mRNA levels (Figure 3A). Conversely, small interfering RNA (siRNA)-mediated *Crls1* knockdown in brown and white thermogenic adipocytes significantly reduced NE-induced uncoupled respiration (Figures 3C, 3D, and S3A–S3F) and decreased *Ucp1* expression in brown fat cells (Figure S3F). Surprisingly, acute *Crls1* deficiency had little impact on other parameters of mitochondrial respiration, including basal, coupled, and maximal oxygen consumption (Figures 3C and 3D), which could be due to the long half-life of CL.

### Cardiolipin Is Essential for Acute and Adaptive Features of the Adipose Thermogenic Program

**Figure 4:**
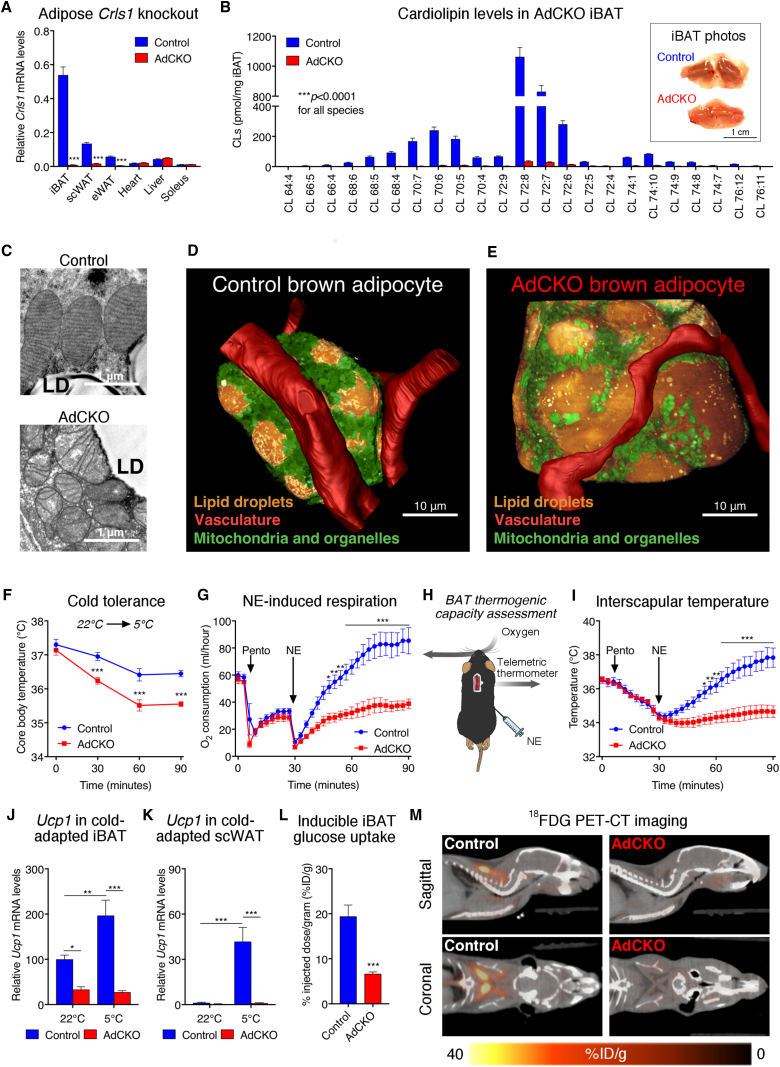
CRLS1 Is Critically Required for the Adipose Thermogenic Program (A) *Crls1* mRNA levels in tissues from control and AdCKO mice (n = 6–7; t tests). (B) Liquid chromatography-mass spectrometry (LC-MS) based quantification of cardiolipin species from control and AdCKO iBAT (n = 6 per group, t tests) and photos of control and AdCKO iBAT (B; inset). (C) Representative transmission electron microscopy (TEM) images of control and AdCKO mitochondrial structure. (D and E) Three-dimensional reconstructions of a (D) control and (E) AdCKO adipocyte generated from dual-beam focused ion beam scanning electron microscopy stacks. (F) Rectal temperature of control and AdCKO mice subjected to a 22°C–4°C cold challenge for 1.5 hr (n = 8 per group; two-way ANOVA). (G–K) (G) Oxygen consumption, (H) schematic of BAT thermogenesis assessment, and (I) interscapular temperature from control and AdCKO mice following anesthetization and 1 mg/kg NE subcutaneous administration (n = 6 per group, two-way ANOVA). *Ucp1* mRNA in (J) iBAT and (K) scWAT from control and AdCKO mice before and after 3 weeks of cold adaptation. (L and M) (L) Quantified mean glucose uptake and (M) representative images of ^18^F-fluorodeoxyglucose positron emission tomography/computed tomography (FDG-PET/CT) from control and AdCKO mice following intraperitoneal administration of CL-316,243. Data are presented as means ± SEM. ^∗^p < 0.05; ^∗∗^p < 0.01; ^∗∗∗^p < 0.001. See also Figures S4 and S5 and Videos S1, S2, S3, and S4.

To investigate the role of adipose CL *in vivo*, we generated a conditional knockout mouse model targeting exon 4 of the *Crls1* gene (*Crls1*^*f/f*^). Tamoxifen (TAM)-induced deletion of *Crls1* exon 4 in primary brown adipocytes reduced CL synthesis and decreased inducible uncoupled respiration, mirroring our siRNA results (Figures 3E, 3F, and S3H). The acute nature of *Crls1* knockdown in our cell models paired with the slow turnover of CLs could account for the modest inhibition of uncoupled respiration observed *in vitro*. To generate constitutive adipose-specific *Crls1* knockout (AdCKO) mice (Figure 4A), we crossed *Crls1*^*f/f*^ mice with animals expressing Cre recombinase under the control of the adiponectin promoter (Eguchi et al., 2011). *Crls1* mRNA and protein were nearly abolished across adipose depots in AdCKO mice (Figures 4A and S4A). Mass spectrometry-based lipidomics of AdCKO iBAT revealed a virtual ablation of all CL species (Figure 4B), resulting in AdCKO iBAT that was distinctly pale compared with controls (Figure 4B, inset).

The AdCKO iBAT expression profile of genes linked to thermogenesis, adipogenesis, and mitochondrial respiration more closely resembled that of white fat than brown (Figures S4B and S4C). Brown adipose from AdCKO mice also had reduced mitochondrial mass (Figures S4D), disrupted cristae structure (Figure 4C), altered respiratory chain complex and supercomplex formation (Figure S4E), and reduced GDP-inhibitable (UCP1-linked) respiration in isolated mitochondria (Figure S4F). The mitochondrial dysfunction in AdCKO iBAT resulted in distorted cellular and lipid droplet morphology (Figures 4D, 4E, and S5A–S5D; Videos S1 and S2). Interestingly, loss of *Crls1* was not as detrimental to white adipose depots (Figures S4B, S4C, and S5C). Although mtDNA content was markedly decreased in AdCKO brown fat, mtDNA levels in epididymal white adipose tissue (eWAT) were unchanged (Figure S5D).

We next assessed the role of adipose CL in body temperature defense during acute cold exposure. Cold tolerance was substantially reduced in AdCKO mice (Figure 4F). To specifically interrogate thermogenic fat activity, NE was administered to anesthetized mice at thermoneutrality. Whereas NE robustly induced oxygen consumption and heat production in controls, these effects were largely abolished in AdCKO mice (Figures 4G–4I). These effects were more dramatic than those observed in our cellular loss-of-function studies, likely due to the more complete loss of CLs in our *in vivo* model. In addition, cold exposure did not induce Ucp1 transcription in iBAT or scWAT of AdCKO mice (Figures 4J and 4K).

Another characteristic feature of mouse and human thermogenic fat is a profound ability to consume glucose from the blood in response to cold or β3-adrenergic stimulation (Cannon and Nedergaard, 2004, Cypess et al., 2009, van Marken Lichtenbelt et al., 2009, Saito et al., 2009, Virtanen et al., 2009). To test the impact of CL on glucose uptake *in vivo*, we measured the inducible accumulation of ^18^F-fluorodeoxyglucose (FDG) into iBAT by positron emission tomography/computed tomography (PET/CT). Animals were injected with the β3 agonist CL-316,243 to specifically stimulate glucose uptake into thermogenic fat. Loss of adipose CL reduced brown fat glucose uptake compared with controls (Figures 4L and 4M; Videos S3 and S4). These collective findings show that CL integrally shapes both acute and adaptive thermogenic responses and inducible glucose uptake in fat.

### Mitochondrial CL Deficiency Suppresses Nuclear-Encoded Genes through the Endoplasmic Reticulum Stress Response Factor CHOP-10

**Figure 5:**
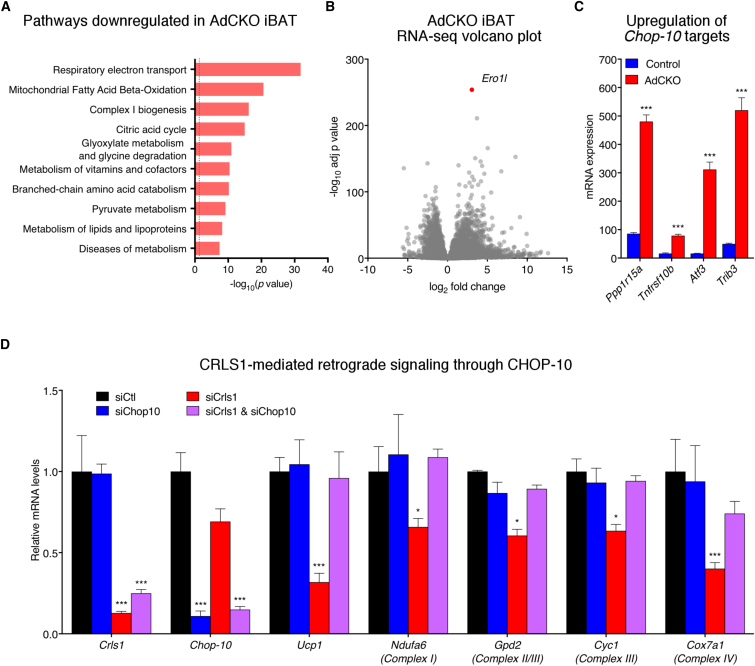
Mitochondrial CL Deficiency Suppresses Nuclear-Encoded Genes through the ER Stress Response Factor CHOP-10 (A) Reactome pathways downregulated in AdCKO iBAT compared with control (RNA sequencing [RNA-seq] data; n = 5 per group; housed at 22°C; dashed line indicates adjusted p value <0.05). (B) Volcano plot of RNA-seq data from AdCKO and control iBAT (n = 5 per group). (C) mRNA levels of known CHOP-10 targets from RNA-seq data (n = 5 per group). (D) mRNA levels from brown adipocytes treated with control, *Crls1*, *Chop10*, or both *Crls1* and *Chop10* siRNA. Data are presented as means ± SEM. ^∗^p < 0.05; ^∗∗∗^p < 0.001.

CL is well established as a direct modulator of mitochondrial structure and bioenergetic capacity (Houtkooper and Vaz, 2008, Paradies et al., 2014). However, our observations revealing *Crls1*-dependent regulation of *Ucp1* transcription in both cells and tissue (Figures 3A, 3B, S3F, and S4B) suggest an additional role for CLs in retrograde communication from the mitochondria to the nucleus. This phenomenon is unlikely due to general mitochondrial dysfunction as other models in which adipose mitochondria are severely disrupted do not result in altered *Ucp1* expression (Ryu et al., 2013, Vernochet et al., 2014). Comparison of AdCKO and control iBAT transcriptomes revealed that CL depletion globally decreased oxidative phosphorylation and other mitochondrial gene programs (Figure 5A).

To gain insight into the potential mechanism through which this regulation was occurring, we examined genes that were upregulated by loss of CL. The gene most significantly increased in AdCKO iBAT over controls was *Ero1l*, an established endoplasmic reticulum (ER) stress response gene (Figure 5B). *Ero1l* is potently activated by the transcriptional regulator, C/EBP homologous protein 10 (CHOP-10, also known as DDIT3 or GADD153) (Marciniak, 2004). Several other genes activated by CHOP-10 were similarly increased in AdCKO iBAT (Figure 5C). Interestingly, CHOP-10 not only activates ER stress response genes but can also directly suppress C/EBP target genes (Oyadomari and Mori, 2004), including those involved in adipose differentiation (Batchvarova et al., 1995). Whether CHOP-10 modulates thermogenic fat gene expression downstream of CL is unknown. Strikingly, the reduction of *Ucp1* and other nuclear-encoded mitochondrial genes caused by loss of *Crls1* was completely blocked by *Chop-10* knockdown in brown adipocytes (Figure 5D). Thus, we have uncovered a novel role for adipose mitochondrial CL in modulating nuclear transcription via the ER stress response factor, CHOP-10.

### Thermogenic Fat Cardiolipin Controls Systemic Metabolic Flexibility and Glucose Homeostasis

**Figure 6:**
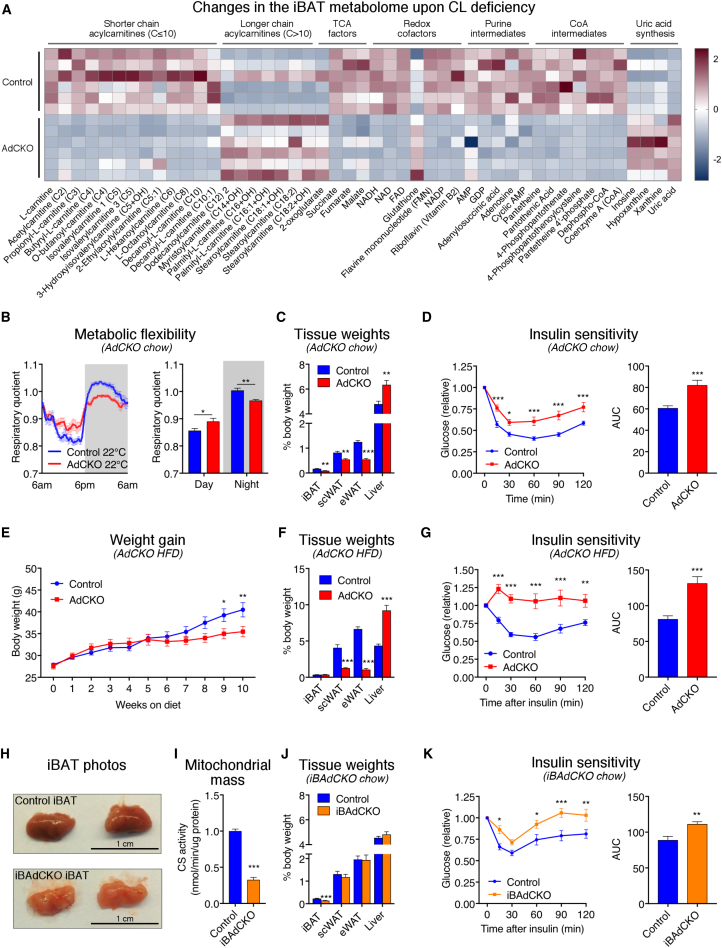
CL Depletion in Fat Promotes Whole-Body Metabolic Inflexibility and Insulin Resistance (A) Heatmap of significantly changed metabolites from targeted LC-MS metabolomics of iBAT from control and AdCKO mice (n = 6 per group, t tests, p < 0.05). (B) Daily RQ for control and AdCKO mice housed in metabolic cages (n = 6 per group). (C and D) (C) Tissue weights (n = 7–10 per group; t tests), and (D) insulin sensitivity of control and AdCKO mice on chow diet (n = 11 per group, two-way ANOVA for x-y plots and t test for area under the curve [AUC]). (E–G) (E) Weight gain, (F) tissue weights, and (G) insulin sensitivity of control and AdCKO mice on HFD (n = 11 per group, two-way ANOVA for x-y plots and t test for AUC). (H) Photos of control and iBAdCKO iBAT tissues. (I–K) (I) Mitochondrial content (measured by citrate synthase [CS] activity; n = 8 per group, t tests), (J) tissue weights (n = 8 per group, t tests), and (K) insulin sensitivity of control and iBAdCKO mice fed chow diet (n = 7 per group; two-way ANOVA for x-y plots and t test for AUC). Data are presented as means ± SEM. ^∗^p < 0.05; ^∗∗^p < 0.01; ^∗∗∗^p < 0.001. See also Figure S6.

Given the profound impact of CL on mitochondrial function, glucose uptake, and nuclear transcription in thermogenic fat, we next investigated the role of CL biosynthesis in both brown adipose and organismal metabolic homeostasis. CL depletion in iBAT created a pathological metabolite signature underscored by a shift in acylcarnitines from shorter (≤10 carbons) to longer chain species (>10 carbons) and significant decreases in tricarboxylic acid (TCA) intermediates, redox cofactors, uric acid production, purine nucleotides, and coenzyme A-related metabolites (Figures 6A and S6A).

We used indirect calorimetry to evaluate the consequence of adipose *Crls1* deficiency on systemic energy homeostasis. AdCKO mice exhibited a marked blunting in the daily respiratory quotient (RQ) biorhythm compared with controls indicating reduced metabolic flexibility (Figure 6B). Furthermore, AdCKO mice on chow diet displayed significantly reduced fat accumulation in adipose depots (Figure 6C) and decreased insulin sensitivity (Figures 6D and S6B) compared with controls despite having no genotypic differences in body weight or composition, food intake, physical activity, or energy expenditure (Figures S6C–S6F). AdCKO livers were larger than controls but hepatic triglyceride concentrations were unchanged (Figure S6G). When challenged on a 60% high-fat diet (HFD), AdCKO mice gained significantly less weight and had more lean mass and less fat mass than controls (Figures 6E and S6H). HFD exacerbated the chow-fed AdCKO phenotype in adipose and liver sizes (Figure 6F), increased fasting blood glucose (Figure S6I), and rendered AdCKO mice completely refractory to insulin (Figure 6G). While the precise mechanism(s) of how adipose CL depletion has such profound ability to modulate organismal energy homeostasis is unknown, one potential contribution may come from signaling factors released from the fat tissue into circulation. Indeed, AdCKO iBAT displayed significant increases in the expression of several secreted factors (Figure S6J) that are documented effectors of systemic metabolism (Villarroya et al., 2016).

While the AdCKO model demonstrates the importance of adipose CL in glycemic control, it cannot distinguish the contribution of thermogenic adipocytes from that of white. Therefore, we generated inducible brown and beige adipose *Crls1* knockouts (iBAdCKO) by crossing *Crls1*^*f/f*^ mice with animals expressing a Cre recombinase and estrogen receptor fusion protein under the control of the *Ucp-1* promoter (Rosenwald et al., 2013) (Figure S6K). Induced *Crls1* deletion in iBAT of adult mice led to distinctly paler brown fat (Figure 6H), decreased nuclear-encoded mitochondrial genes in iBAT (Figure S6L), and reduced iBAT mitochondrial mass (Figures 6I and S6M), consistent with the results from AdCKO mice. In contrast to AdCKO mice, iBAdCKO mice showed no changes in expression of mitochondrial genes in scWAT (Figure S6N), nor did they exhibit fat loss in white adipose depots or hepatomegaly (Figure 6J). The difference in white fat weights in AdCKO but not iBAdCKO mice highlights the importance of mitochondria for the integrity and storage capacity of white adipose tissue. Notably, iBAdCKOs still displayed marked insulin resistance (Figure 6K). Thus, our results reveal that CL in thermogenic fat mitochondria exerts profound control over whole-body energy homeostasis independent of changes in white adipose tissue.

### Adipose *CRLS1* Is Positively Linked to Insulin Sensitivity in Humans and Boosts Fat Cell Respiration

**Figure 7:**
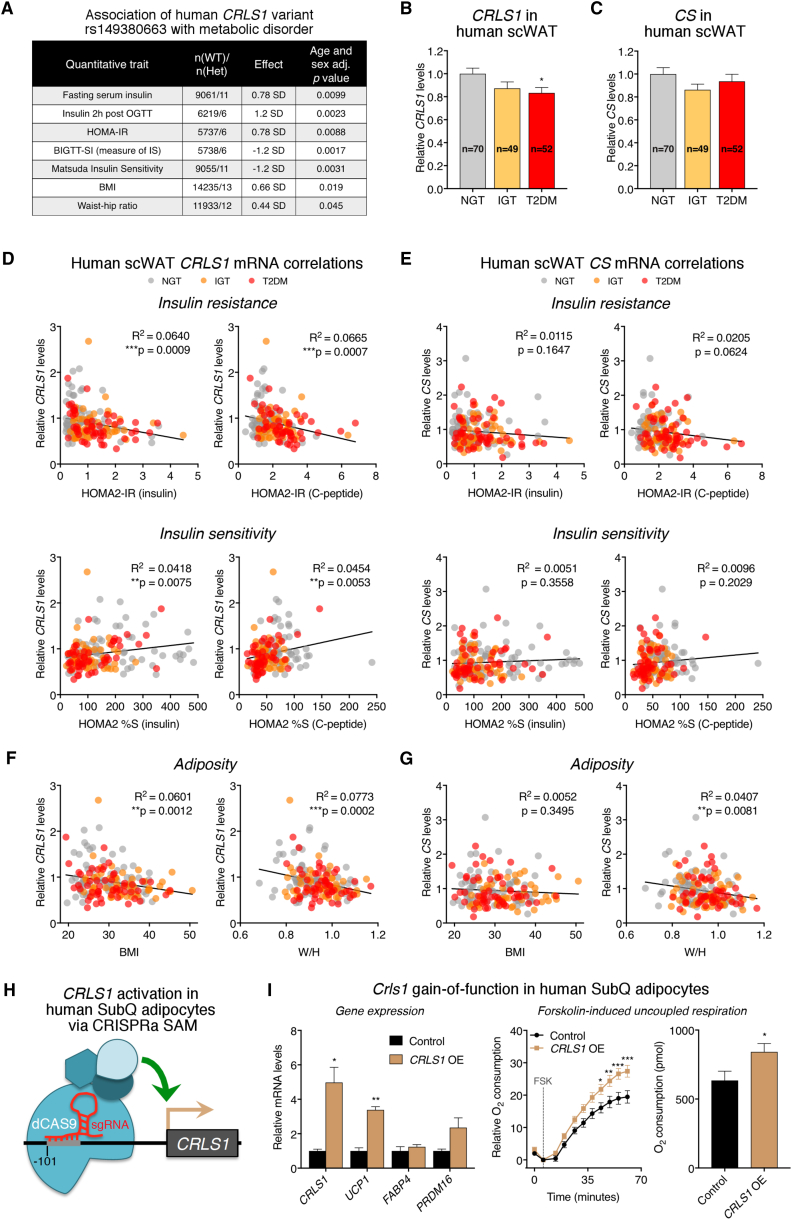
Adipose *CRLS1* Is Linked to Whole-Body Insulin Sensitivity in Humans and Augments Human Fat Cell Energy Expenditure (A) Genetic link between a rare *CRLS1* variant and metabolic disease from Danish population-based cohorts. (B–G) *CRLS1* (B) and *CS* (C) mRNA levels in the scWAT of normal glucose tolerant (NGT), impaired glucose tolerant (IGT), and type 2 diabetic mellitus (T2DM) donors (n = 70, 49, and 52 per group, respectively, one-way ANOVA). Pearson correlations between scWAT *CRLS1* (D) and *CS* (E) *mRNA* levels and homeostatic model assessment two insulin resistance (HOMA2-IR, calculated by insulin and c-peptide), HOMA2 insulin sensitivity (%S, calculated by insulin and c-peptide) and adiposity parameters (F and G). Pearson R^2^ values and significance are shown. W/H, weight/height. (H) Schematic for CRISPRa synergistic activation mediator (SAM) targeting of *CRLS1* in human white adipocytes. (I) Gene expression and forskolin (Fsk)-induced respiration (t test) from human white adipocytes transduced with empty vector or vector delivering a single-guide RNA directed to the −101 position upstream of the *CRLS1* transcriptional start site. Data are presented as means ± SEM. ^∗^p < 0.05; ^∗∗^p < 0.01; ^∗∗∗^p < 0.001. See also Figure S6 and Table S1.

To address the clinical relevance of our findings, we analyzed genetic associations from several Danish population-based cohorts for links between CL-related genes and parameters of metabolic disease. We identified a rare (minor allele frequency 0.05%) synonymous variant in *CRLS1* (rs149380663) that is positively associated with multiple indicators of insulin resistance and negatively associated with measures of insulin sensitivity (Figure 7A). Conversely, no such associations were found with SNPs for other enzymes involved in the *de novo* CL synthesis pathway (Table S1). Given the enrichment of *CRLS1* in human brown fat, it is tempting to speculate that adipose CL biosynthesis is, at least in part, contributing to the observed association with glycemic control. However, genetic variants will potentially have an impact on any tissue that expresses *CRLS1*, thus it is not currently possible to assign causality of this variant to fat.

Therefore, we directly investigated whether altered *CRLS1* levels in human adipose tissue were linked to metabolic disease. Subcutaneous adipose tissue was analyzed from 171 subjects with normal glucose tolerance (NGT), impaired glucose tolerance (IGT), or type 2 diabetes mellitus (T2DM). We found that expression of *CRLS1* mRNA was significantly reduced in subcutaneous fat from diabetic subjects (Figure 7B). Importantly, this decrease in *CRLS1* in T2DM patients was not attributable to a general trend in mitochondria-linked gene expression, as mRNA levels of the canonical mitochondrial marker citrate synthase (*CS*) were unchanged across disease status (Figure 7C). We then assessed the relationship between *CRLS1* and *CS* mRNA levels and several related parameters of glucose metabolism and adiposity. The *CRLS1* mRNA levels in human scWAT negatively correlated with HOMA2 measures of insulin resistance and positively correlated with HOMA2 measures of insulin sensitivity (Figure 7D). Conversely, no such association was found with *CS* (Figure 7E). However, both *CRLS1* and *CS* negatively correlated with waist/hip ratio (Figures 7F and 7H). These findings suggest that *CRLS1* has a unique influence on systemic glycemic control.

To determine if the positive link between adipose *CRLS1* and metabolic homeostasis in humans could be partly attributable to cell-autonomous effects, we performed *CRLS1* gain-of-function studies in human white adipocytes isolated from the neck region. We used a CRISPRa synergistic activation mediator (SAM) system (Lundh et al., 2017) to drive expression of the endogenous *CRLS1* gene (Figure 7H). As in our mouse brown adipocytes, increased *CRLS1* expression in human white adipocytes stimulated *UCP1* expression and increased inducible uncoupled respiration (Figures 7I and S6O). Taken together, these data show that CRLS1 is significantly linked to insulin sensitivity in humans and can be targeted to stimulate energy expenditure in adipocytes.

## Discussion

Products of lipid metabolism, including fuels, signaling molecules, and cellular structural components, are broadly required for activation of thermogenic adipocytes (Cannon and Nedergaard, 2004, Lynes et al., 2017, Ouellet et al., 2012, Simcox et al., 2017), yet little is known about which specific lipid pathways are most influential in shaping thermogenic fat function. Previous snapshots of cold exposed brown fat have indicated the importance of TAG and phospholipid metabolism (Forner et al., 2009, Marcher et al., 2015). In the present study, we generated the first global proteomic and lipidomic landscapes of brown fat throughout the dynamic adaptation to cold temperature. Combined with beige fat lipidomics, we were able to pinpoint the mitochondrial inner membrane phospholipid, CL, and its synthase, CRLS1, as primary mediators in the activation and recruitment of thermogenic fat.

We found *Crls1* to be overwhelmingly enriched in iBAT compared with other major metabolic tissues. Loss of *Crls1* and CL abolished the thermogenic capacity of adipose tissue through a dramatic disruption of mitochondrial bioenergetics. In addition to ubiquitous roles in the formation and maintenance of mitochondrial function, CL is likely to have brown and beige fat-specific functions in thermogenesis. One such function could be direct activation of the thermogenic effector, UCP1. Previous *in vitro* studies have demonstrated that CL binds tightly to UCP1, aids in its proper folding (Hoang et al., 2013, Lee et al., 2015), and decreases its binding affinity for inhibitory purine nucleotides (Klingenberg, 2009). Moreover, one of the predicted CL binding sites on UCP1 is in close proximity to a cysteine residue whose free radical-induced sulfenylation promotes thermogenic capacity (Chouchani et al., 2016). In addition, CL physically interacts with creatine kinase (Paradies et al., 2014), a key enzyme that drives a heat-producing futile cycle in beige fat (Kazak et al., 2015). Therefore, it is likely that CRLS1 and CL synthesis are central to both UCP1-dependent (Chouchani et al., 2016, Golozoubova et al., 2001, Shabalina et al., 2013) and -independent (Kazak et al., 2015) thermogenic mechanisms.

Our studies unexpectedly led to the discovery of a novel role of CL in mitochondria-to-nucleus retrograde communication. Loss of CL synthesis caused global, cell-autonomous changes in nuclear transcription that included a repression of *Ucp1* and mitochondrial gene programs. This transcriptional control was mediated through the ER stress response factor CHOP-10. Intriguingly, *Crls1* gain-of-function increased *Ucp1* expression and inducible uncoupled respiratory capacity in both mouse and human adipocytes. The mechanism underlying this positive feedforward activation of the thermogenic program by CL synthesis in adipose tissue is not clear. Nevertheless, the dynamic nature of CRLS1 mitonuclear signaling suggests that CL may serve as a rheostat to convey the mitochondrial bioenergetic potential to the nucleus in order to coordinate maximal thermogenic capacity.

The activity of thermogenic adipose tissue is strongly correlated with parameters of metabolic health (Lee et al., 2013) and positively modulates glucose homeostasis in mice (Stanford et al., 2013) and humans (Chondronikola et al., 2014), yet until recently, it was unknown if dysfunction of adipose thermogenesis could contribute to insulin resistance. The advent of brown and beige adipocyte-specific loss-of-function models (Kong et al., 2014, Rosenwald et al., 2013) has prompted conceptual leaps in the understanding of how thermogenic fat cells influence systemic metabolism. For example, deletion of the transcriptional regulator *Irf4* in *Ucp1*^+^ adipocytes disrupts the thermogenic gene program and exacerbates HFD-induced insulin resistance (Kong et al., 2014). Surprisingly, several studies have ablated major cellular programs in *Ucp1*^*+*^ adipocytes, including AKT2 signaling (Sanchez-Gurmaches et al., 2018), ATGL-mediated lipolysis (Schreiber et al., 2017), and mitochondrial fusion/fission dynamics (Mahdaviani et al., 2017), without reducing glycemic control. In contrast to these findings, we demonstrate for the first time that mitochondrial dysfunction in thermogenic fat is sufficient to cause whole-body insulin resistance.

Disruption of CL species is implicated in the pathology of several diseases, notably Barth syndrome, cardiomyopathy, metabolic disease, and cancer (Houtkooper and Vaz, 2008, Shi, 2010). Our tissue-specific *Crls1* knockout models provide definitive causality between deficient CL synthesis in thermogenic fat and diabetic pathogenesis. Our discoveries of a *CRLS1* variant associated with insulin resistance and reduced *CRLS1* levels in fat from diabetic patients indicate that CL biosynthesis in adipose tissue could contribute to glycemic balance in humans. Importantly, we found a positive correlation between adipose *CRLS1* levels and clinical markers of insulin sensitivity and showed that induction of *CRLS1* expression in human adipocytes can increase energy dissipation. These findings suggest that positive modulation of CLs in fat could improve glucose homeostasis and promote metabolic fitness. Taken together, our data support a novel paradigm in which adipose CL synthesis is a key control point for systemic glucose regulation.

### Limitations of the Study

The use of Adipoq-Cre in a majority of our studies makes it difficult to dissect specific effects of CLs in white adipose tissue versus BAT. To address brown fat contributions, we generated an inducible BAT-specific Crls1 knockout model, iBAdCKO, yet the specific effect of CL depletion in white adipose remains to be determined. Another limitation is that, while we have clearly shown mechanistic data from cells that reduced CL levels suppress *Ucp1* expression through the ER stress response protein CHOP-10, we do not know the signaling molecules that trigger this effect. Our data from human adipose tissue are limited to mRNA levels and would be strengthened if we could quantify the levels of CLs within these or similar samples. Finally, a weakness of our human data is that the *CRLS1* SNP we analyzed is non-coding, and we have not investigated the effect of this mutation on gene expression or function. We hope that future work will provide a greater understanding of how CLs modulate systemic metabolism through adipose tissue mitochondria.

## STAR★Methods

### Key Resources Table

**Table:** 

REAGENT or RESOURCE	SOURCE	IDENTIFIER
**Antibodies**		

Rabbit polyclonal anti-CRLS1	This paper	N/A
Donkey ECL Rabbit IgG HRP-linked whole antibody	GE Healthcare	CAT#NA934; RRID: AB_772211
anti-OxPhos antibody cocktail	Life Technologies	CAT#457999; RRID: AB_2533834
anti-MTCO1	Abcam	CAT#ab14705; RRID: AB_2084810

**Biological Samples**		

Human supraclavicular BAT and subcutaneous adipose tissue samples	Jespersen et al., 2013	N/A
Human subcutaneous abdominal tissue samples	Pedersen et al., 2012	N/A

**Chemicals, Peptides, and Recombinant Proteins**		

Collagenase I (mouse and human primary cultures)	Worthington Biochemical	CAT#LS004194
Bovine serum albumin (respirometry and mouse primary culture)	Sigma Aldrich	CAT#A7030
Bovine serum albumin (human primary cultures)	Worthington Biochemical	CAT#LS000290
DMEM, high glucose GlutaMAX (mouse adipocyte cultures)	Thermo fisher	CAT#31966021
Basal DMEM for Seahorse respirometry	Sigma Aldrich	CAT#D5030
Fetal bovine serum (mouse adipocyte cultures)	Sigma Aldrich	CAT#F7524
Penicillin/streptomycin (mouse adipocyte cultures)	Lonza	CAT#DE17-602E
Dexamethasone	Sigma Aldrich	CAT#D4902
Rosiglitazone	Cayman	CAT#71740-5
T3	Sigma Aldrich	CAT#T6397
Insulin	Sigma Aldrich	CAT#I9278
3-Isobutyl-1-methylxanthine (IBMX)	Sigma Aldrich	CAT#I5879
DMSO	Sigma Aldrich	CAT#D5879
PhosSTOP	Sigma Aldrich	CAT#04906845001
Protease inhibitor cocktail	Promega	CAT#6521
LysC endocpeptidase (Lyso endopeptidase)	Wako	CAT#125-05061
Trypsin	Promega	CAT#V5280
TMT10-plex isobaric label reagent set	Thermo Scientific	CAT#90110
Precellys lysing kit	Bertin Corp, Precellys	CAT#KT03961-1-001.2
Tamoxifen (for in vivo experiments)	Sigma Aldrich	CAT#T5648
Corn oil	Sigma Aldrich	CAT#C8267
Tri reagent	Sigma Aldrich	CAT#T9424
5% Digitonin	Novex	CAT#BN2006
NativePAGE Sample Buffer	Novex	CAT#BN20032
NativePAGE Coomassie G-250	Novex	CAT#BN2004
NativePAGE Running Buffer	Novex	CAT#BN2001
Coomassie Brilliant Blue G-250	SERVA	CAT#17524
NativePAGE 3-12% Bis-Tris Protein Gels	Novex	CAT#BN2011BX10
Western Breeze Chromogenic Immunodetection System	Novex	CAT#WB7103
Infinity Triglyceride Liquid Stable Reagent	Thermo Scientific	CAT#TR22421
Pierce Protease and Phosphatase Inhibitor Mini Tablets	Thermo Scientific	CAT#A32959
Nupage LDS Sample Buffer	Thermo Scientific	CAT#NP0008
NuPAGE 3-8% Tris-Acetate Protein Gels	Thermo Scientific	CAT#EA0375BOX
ECL Rabbit IgG, HRP-linked whole Ab	GE Healthcare Life Sciences	CAT#NA934
4-hydroxytamoxifen (for in vitro experiments)	Sigma	CAT#H6278
Lipofectamine RNAiMAX Transfection Reagent	Thermo Scientific	CAT#13778100
SuperFect Transfection Reagent	Qiagen	CAT#301305
Lenti-X Concentrator	Clontech	CAT#631231
Oligomycin	Cayman Chemical	CAT#11342
(±)-Norepinephrine (+)-bitartrate salt (NE)	Sigma Aldrich	CAT#A0937
Trifluoromethoxy carbonylcyanide phenylhydrazone (FCCP)	Cayman Chemical	CAT#15218
Rotenone	Sigma Aldrich	R8875
Antimycin A	Sigma Aldrich	A8674
Oxaloacetic acid	Sigma Aldrich	CAT#04126
Acetyl-CoA	Sigma Aldrich	CAT#A2182
Lipid internal standards	Various	See Lipidomics section of STAR Methods

**Critical Commercial Assays**		

BCA protein assay kit	Thermo Scientific	CAT#23225
Micro BCA protein assay kit	Thermo Scientific	CAT#23235
Cell Line Nucleofector Kit L	Lonza	CAT#VACA-1005
PolyJet DNA *in vitro* transfection reagent	SignaGen	CAT#SL100688
Gene Elute Mammalian DNA Miniprep Kit	Sigma Aldrich	CAT#G1N70
Seahorse XFe96 FluxPax	Agilent	CAT#102416-100
High-Capacity cDNA Reverse Transcription Kit	Thermo Scientific	CAT#4368814
Direct-zol RNA MiniPrep kit	Zymo Research	CAT#R2071
Affymetrix GeneChip 3’IVT Express Kit	Thermo Scientific	CAT#902416

**Deposited Data**		

iBAT RNA-seq data from Control and AdCKO mice housed at 22°C	This paper	GEO: GSE110297

**Experimental Models: Cell Lines**		

Immortalized mouse brown preadipocytes	Harms et al., 2014	N/A
CRISPRa *CRLS1* gain-of-function immortalized subcutaneous neck fat preadipocytes	This paper	N/A
Phoenix-A cells	ATCC	CAT#CRL-3213

**Experimental Models: Organisms/Strains**		

Mouse: Male wild-type C57Bl/6NRj	Janvier	N/A
Mouse: Floxed (exon 4) *Crls1* mice (*Crls1*^*f/f*^)	This paper	N/A
Mouse: Adipoq-Cre mice, B6;FVB-Tg(Adipoq-cre)1Evdr/J	Jackson Laboratories; Eguchi et al., 2011	Stock No: 028020
Mouse: Rosa26ERT2, B6.129-*Gt(ROSA)26Sor*^*tm1(cre/ERT2)Ty*^*j*/J	Jackson Laboratories	Stock No: 008463
Mouse: Ucp1-Cre mice, B6-Tg(Ucp1-cre/ERT2)426Biat	Rosenwald et al., 2013	N/A

**Oligonucleotides**		

siRNA Control	Sigma	SIC001
siRNA targeting *Crls1*	Sigma	SASI_Mm01_00094096
siRNA targeting *Ddit3* (*Chop10*)	Sigma	SASI_Mm01_00085431
gRNA for *CRLS1* CRISPRa gain-of-function (CATCAGGCTCAGTGGGTTTT)	This Paper	N/A
RT qPCR Primers	This Paper	See Table S2

**Recombinant DNA**		

pBABE-hTERT-Hygro	Addgene	CAT#1773
pcDNA3.1+/C-(K)DYK-*Crls1*	Addgene	OMu17939

**Software and Algorithms**		

Graphpad Prism 7.0 for statistical analysis	GraphPad	N/A

**Other**		

Danish Population-based cohorts for genetic variant analysis: Inter99, Health2006, Health2008, Vejle Biobank and type 2 diabetes patients from Steno Diabetes Center	Albrechtsen et al., 2013, Jørgensen et al., 2003, Thuesen et al., 2014	N/A

### Contact for Reagent and Resource Sharing

Further information and requests for resources and reagents should be directed to and will be fulfilled by the Lead Contact, Zachary Gerhart-Hines (zpg@sund.ku.dk).

### Experimental Model and Subject Details

#### Wild-Type Mice for Cold Exposure Studies and Primary Cell Culture

All animal studies were performed with approved protocols from the *The Danish Animal Experiments Inspectorate* permit number 2014-15-0201-00181 and the University of Copenhagen project number P14-379 and P16-021. Male wild-type C57Bl/6NRj mice (Janvier) were used for cold exposure studies and primary preadipocyte cultures. Unless otherwise noted, mice were group housed on a 12-hour light/dark cycle (lights on at 6:00 and off at 18:00) and given ad libitum access to chow food (Altromin, 1310). For cold exposure experiments, twelve-sixteen week old mice were individually housed and placed into climate controlled rodent incubators (Memmert HPP750Life) set to 29°C to acclimate to thermoneutrality for two weeks. Cold exposure mice were then moved to an incubator set to 5°C and kept there until dissection.

#### Genetically-Modified Mouse Lines

Floxed *Crls1* mice (*Crls1*^*f/f*^) were generated by Genoway (France). Exon 4, which contains part of the CDP-alcohol phosphatidyltransferase domain, was flanked by *loxP* sites. Cre-mediated deletion of exon 4 disrupts the enzymatic domain of CRLS1 and results in out-of-frame splicing and premature stop codons. To achieve adipose specific or tamoxifen inducible deletion, floxed models were crossed with Adipoq-Cre mice, B6;FVB-Tg(Adipoq-cre)1Evdr/J (Jackson Laboratories Stock No: 008463) (Eguchi et al., 2011) or Rosa26ERT2, B6.129-*Gt(ROSA)26Sor*^*tm1(cre/ERT2)Ty*^*j*/J (Jackson Laboratories Stock No: 008463). For studies with Adipoq-Cre/*Crls1*^*f/f*^ mice, controls were homozygous floxed without Cre. For primary cell studies using tamoxifen to induce *Crls1* knockout using Rosa26ERT2/*Crls1*^*f/f*^ mice, controls were homozygous floxed without Cre treated with tamoxifen. For *Crls1* knockout in UCP1-positive cells, *Crls1*^*f/f*^ mice were crossed with the tamoxifen-inducible B6-Tg(Ucp1-cre/ERT2)426Biat mouse line (kindly provided by Prof. Christian Wolfrum) (Rosenwald et al., 2013). To induce knockout in this model, 2 mg of tamoxifen (Sigma-Aldrich T5648) in 100 μl of corn oil (Sigma-Aldrich C8267) was delivered by oral gavage once per day for 3 consecutive days. Control mice were *Crls1*^*f/f*^ without Ucp1-Cre/ERT2 and were given an identical dosage of tamoxifen.

#### Human Adipose Samples

Samples of human supraclavicular BAT and subcutaneous adipose tissue samples were from a previously published study (Jespersen et al., 2013). Subjects who were suspected of cancer in the neck area were included via the outpatient clinic at the Department of Oto-Rhino-Laryngology Head & Neck Surgery at Rigshospitalet, Copenhagen, prior to elective surgery in the neck area. Control samples originated from seven age, sex and BMI matched participants in the study “Estrogen Receptors and Inflammatory Markers in Adipose Tissue and Muscle. Association between Gender, Adiposity and Insulin Resistance” and were obtained from subcutaneous adipose tissue during cholecystectomy at the Department of Abdominal Surgery, Hvidovre Hospital. Biopsies were obtained during surgery by an experienced surgeon. Tissue was removed using scalpel and scissor. Immediately after removal samples were flash frozen in liquid nitrogen before being stored on – 80°C until analyses were performed. Due to large variation in *UCP1* expression, supraclavicular samples were divided into a BAT high and a BAT low group based on UCP1 expression, n = 10 and n=9 respectively.

Subcutaneous abdominal adipose tissue samples from individuals with normal glucose tolerance (NGT), impaired glucose tolerance (IGT) or type II diabetes similarly originated from a previous study (Pedersen et al., 2012). Biopsies were obtained using a modified version of the Bergström needle biopsy procedure. Participants were classified as having NGT, IGT, or type 2 diabetes according to the definition from the World Health Organization (WHO) on the basis of blood glucose levels while fasting and at 2 h post glucose load during an oral glucose tolerance test (OGTT). The homeostatic model assessment of insulin resistance 2 (HOMA-IR) index for insulin resistance and the estimates of insulin sensitivity (HOMA_%S) were calculated from fasting glucose and insulin, as well as fasting glucose and C-peptide blood concentrations using the HOMA2 Calculator 2.2.3 (Diabetes Trials Unit, University of Oxford, http://www.dtu.ox.ac.uk/homa). The study protocols were approved by The Scientific-Ethics Committees of the Capital Region and of Copenhagen and Frederiksberg Municipalities, Denmark, (journal number H-A-2009-020, H-A-2008-081) and the Regional Committee on Biomedical Research Ethics in Denmark (journal number H-C-2008-101), respectively. All subjects provided written informed consent prior to participation. The Scientific-Ethics Committees of the Capital Region and of Copenhagen and Frederiksberg Municipalities, Denmark approved the study protocols, journal number H-A-2009-020, H-A-2008-081 respectively, and the studies were performed in accordance with the Helsinki declaration.

#### Human Adipocyte Isolation, Immortalization and Culture

For CRISPRa *CRLS1* gain-of-function in human adipocytes, isolation of primary stromal vascular fraction (SVF) cells from human neck fat was described previously (Xue et al., 2015). Specifically, subcutaneous and subplatysmal neck fat depots were pooled to generate hWAT-SVF cells. Freshly resected fat depots were collected, minced and digested using collagenase 1 (2 mg/ml in PBS with the addition of 3.5% BSA; Worthington Biochemical Corporation, Lakewood, NJ), and the SVF was isolated. SVF cells were plated and grown in high-glucose Dulbecco’s modified Eagle’s medium (DMEM/H) supplemented with 10% (vol/vol) fetal bovine serum (FBS) and 1% penicillin-streptomycin. For adipocyte differentiation, cells were grown to confluence for 6 days (referred to as ‘day 6’) and then exposed to adipogenic induction mixture in DMEM/H medium containing 0.5 mM isobutylmethylxanthine, 0.1 μM dexamethasone, 0.5 μM human insulin (Sigma Aldrich), 2 nM T3, 30 μM indomethacin, 17 μM pantothenate, 33 μM biotin and 2% FBS for another 12 days (referred to as ‘day 18’). Induction medium was changed every 3 days until cells were collected.

Primary SVF cells were immortalized with hTERT. Briefly, primary SVF isolated from subjects that had undergone four or five population doublings were separately infected with a retrovirus containing the plasmid, pBABE-hTERT-Hygro (Addgene no. 1773, Cambridge, MA), which expresses hTERT driven by a long-terminal-repeat promoter. Phoenix-A cells (ATCC) were transfected with pBABE-hTERT-Hygro DNA using PolyJet DNA *in vitro* transfection reagent (SignaGen Laboratories, Rockville, MD). Culture supernatants containing virus were collected every 24 h after transfection and filtered through a 0.45 μm filter (Fisher Scientific, Pittsburgh, PA). Primary SVF cells from human white fat at 80% confluence were infected with supernatants in the presence of 4 μg/ml Polybrene every day until cells reached 90% confluence. Cells were then treated with hygromycin (concentrations ranging from 100 μg/ml to 400 μg/ml, depending on cell conditions) in DMEM/H medium containing 10% FBS and antibiotics. Once drug selection was finished, the cells were maintained in culture medium with 50 μg/ml hygromycin for 2 weeks.

Immortalized progenitor cells were plated and grown in DMEM/H medium supplemented with 10% FBS (day 0). For adipocyte differentiation, cell were grown for 6 d until reaching confluence (day 6), and then treated with the adipogenic induction medium as described above for 12 d (day 18). We routinely checked for mycoplasma contamination and all the cells used in this study were free of mycoplasma.

#### Human Genetic Association of Variants in CL Synthesis and Remodeling Genes

We investigated associations between coding genetic variants in four CL-related genes (*CRLS1*, *PGS1*, *CDS2*, *PTPMT1*) and metabolic phenotypes in up to 15,840 Danish individuals from five cohorts: Inter99, Health2006, Health2008, Vejle Biobank and type 2 diabetes patients from Steno Diabetes Center. Information on these cohorts has been published previously (Albrechtsen et al., 2013, Jørgensen et al., 2003, Thuesen et al., 2014). The study was approved by the Regional Ethical Committee of Copenhagen and is in accordance with the scientific principles of the Helsinki Declaration II. We used genetic variation on the Illumina Exome BeadArray v1.0. Genotypes were called using GenCall applying a custom-made cluster file based on 6,000 samples with high quality data. From the ExomeBeadChip, quality control of samples and variants was done using PLINK and included exclusion of samples showing relatedness (first- and second-degree relatives), extreme inbreeding coefficient (F<0.1 or F>0.1), low call rate (<98%) or mismatch between sex status in phenotype and genotype data. All variants obeyed Hardy Weinberg equilibrium. After QC, 15,840 samples fulfilled quality criteria. We identified 10 variants in three (*CRLS1*, *PGS1*, *PTPMT1*) of these four genes with >10 carriers of the alternative allele among all 15,840 samples and these variants were analyzed in relation to metabolic traits related to insulin resistance. Metabolic traits related to insulin resistance were analyzed. These traits were: body mass index (n=14,684), waist-hip ratio (n=12,380), fasting serum insulin (n=9,072), 2-h serum insulin during an oral glucose tolerance test (n=6,225), together with derived indices of insulin sensitivity: homeostasis assessment of insulin resistance (HOMA-IR) (n=9,066), Matsuda Insulin sensitivity index (n=5,744) and BIGTT-insulin sensitivity (BIGTT-SI) (n=5,744). Association analyses were performed by linear regression adjusted for age, sex and the first four principal components from a principal component analysis. Phenotypes were rank normalized separately in each of the cohorts prior to the analysis to obtain a normal distribution.

#### Mouse Adipocyte Cultures

For gain- and loss-of-function studies with primary mouse adipocytes, iBAT (for brown) or scWAT (for beige-like) was freshly dissected from 5 week old male wild-type C57Bl/6NRj mice (for electroporation and siRNA studies) or RosaERT2-*Cre Crls1*^*f/f*^ (for tamoxifen-inducible knockout). For preadipocyte isolation, adipose depots from 2 mice was combined, finely minced with scissors, digested with DMEM containing 0.2% collagenase type 1 (#LS004194, Worthington Biochemical) and 2% bovine serum albumin (BSA; Sigma Aldrich A7030). Digests were centrifuged, resuspended in DMEM (Sigma Aldrich D5030) with 10% fetal bovine serum (FBS; Sigma-Aldrich F7524) and penicillin/streptomycin (Lonza DE17-602E) and transferred through a 40 μm nylon strainer onto 6-well plates. Cells were rinsed the following day, and media was changed every 2 days. Upon confluency, cells were differentiated with DMEM containing a differentiation cocktail of 86 nM insulin, 0.1 μM dexamethasone, 1 μM rosiglitazone, 1 nM T3 and 250 μM methyl isobutyl xanthine (IBMX). After 2 days, differentiation media was replaced with DMEM containing 10% FBS with 0.5 μg/mL insulin with (brown) or without 1 nM T3 (beige-like). Media was changed every 2 days. Cells were incubated at 37°C with 10% CO_2_. Immortalized mouse brown preadipocytes have been previously described (Harms et al., 2014). Cells were grown with DMEM with 10% fetal bovine serum and penicillin/streptomycin. Upon confluency, cells were differentiated with DMEM containing a differentiation cocktail of 20 nM insulin, 1 μM dexamethasone, 0.5 μM rosiglitazone, 1 nM T3 and 500 μM methyl isobutyl xanthine. After 2 days of differentiation, media was replaced with DMEM with 10% FBS containing 1 nM T3 and 20 nM insulin.

### Method Details

#### Proteomics

Twelve-week old male C57Bl/6N mice were acclimated to thermoneutrality (29°C) and individually housed were subject to cold exposure (5°C) for 8 hours, 1 day, 3 days, 1 week or 3 weeks iBAT was collected at dissection and snap frozen in liquid nitrogen. Brown adipose tissues were mechanically lysed with a homogenizer with 2 mL SDS lysis buffer containing 2.0 % SDS w/v, 150 mM NaCl, PhosStop (Roche, Madison, WI) phosphatase inhibitors, EDTA free protease inhibitor cocktail (Promega, Madison, WI) and 50 mM HEPES, pH 8.5. Lysates were reduced with 5 mM DTT and cysteine residues were alkylated at room temperature with iodoacetamide (14 mM) in the dark as previously described (Huttlin et al., 2010). Protein content was purified by methanol/chloroform extraction. Protein disks were resuspended in 8 M Urea containing 50 mM HEPES (pH 8.5) and concentrations were measured by BCA assay prior to protease digestion. 1 mg of protein lysates were diluted to 4 M urea and digested with LysC (Wako, Japan) in a 1/100 enzyme/protein ratio overnight. Protein extracts were diluted further to a 1.0 M urea concentration and trypsin (Promega, Madison, WI) was added to a final 1/200 enzyme/protein ratio for 6 hours at 37°C. Digests were acidified with 20 uL of 20% formic acid (FA) to a pH ∼ 2 and subjected to C18 solid-phase extraction (SPE) (Sep-Pak, Waters, Milford, MA).

Isobaric labeling of peptides was performed using 10-plex tandem-mass tag (TMT) reagents (Thermo Fisher Scientific, Rockford, IL). TMT reagents (0.8 mg) were dissolved in 42μl dry acetonitrile (ACN) and 10 μl was added to 100 μg of peptides dissolved in 100 μl of 200mM EPPS, pH 8.0. After 1hr (RT), the reaction was quenched by adding 4 μl of 5% hydroxylamine. Labeled peptides were combined, acidified with FA (pH ∼2) and diluted to a final ∼5% ACN concentration prior to C18 SPE on Sep-Pak cartridges (50 mg).

Basic pH reversed-phase HPLC (bpHrp) TMT labeled peptides were subjected to orthogonal bpHrp fractionation. Labeled peptides were solubilized in buffer A (5% ACN 10 mM ammonium bicarbonate, pH 8.0) and separated by an Agilent 300 Extend C18 column (5 μm particles, 4.6 mm ID and 220 mm in length). Using an Agilent 1100 binary pump equipped with a degasser and a photodiode array (PDA) detector (Thermo Scientific, San Jose, CA), a 50 min linear gradient from 12% to 45% acetonitrile in 10 mM ammonium bicarbonate pH 8 (flow rate of 0.8 mL/min) separated the peptide mixtures into a total of 96 fractions. 96 Fractions were consolidation into 12 samples, acidified with 10 μl of 20% formic acid and vacuum dried. Each sample was re-dissolved in 5% formic acid, desalted via StageTips, dried via vacuum centrifugation, and reconstituted for LC-MS/MS analysis.

Mass spectrometry analysis: All bpHrp fractions were subjected to LC-MS/MS analyses onto a LTQ Orbitrap Velos Fusion (Thermo Scientific San José, CA) instrument equipped with a Famos autosampler (LC Packings, Sunnyvale, CA) and an Agilent 1100 binary HPLC pump (Agilent Technologies, Santa Clara, CA). Peptides were separated onto a 100 μm I.D. microcapillary column packed first with approximately 1 cm of Magic C4 resin (5μm, 100 Å, Michrom Bioresources, Auburn, CA) followed by ∼25 cm of Maccel C18AQ resin (1.8 μm, 200 Å, Nest Group, Southborough, MA). Peptides were separated by applying a gradient from 5 to 35% ACN in 0.5% FA over 180 min at ∼200 nl/min. Electrospray ionization implemented through applying a voltage of 1.86 kV using an inert gold electrode via a PEEK junction at the end of the microcapillary column. The LTQ Orbitrap Velos Fusion was operated in data-dependent manner for the MS methods. The MS survey scan was performed in the Orbitrap in the range of 400-1300 m/z at a resolution of 3x104, followed by the selection of the ten most intense ions (TOP 10) for CID-MS2 fragmentation in the ion trap using a precursor isolation width window of 2 m/z, AGC setting of 2000, and a maximum ion accumulation of 150 ms. Normalized collision energy was set to 35% and an activation time of 20ms. Ions within a 10 ppm m/z window around ions selected for MS2 were excluded from further selection for fragmentation for 120s. Directly following each MS2 event, 6-10 of most intense fragment ion in an m/z range between 110-160% of the precursor m/z was selected for HCD-MS3 (McAlister et al., 2012, Ting et al., 2011). The fragment ion isolation width was set to 4 m/z, AGC was set to 20,000, the maximum ion time was 250ms, normalized collision energy was set to 60% and an activation time of 50ms for each MS3 scan. For all MS3 scans, Orbitrap resolving power was set to 30,000 (@ 400 m/z).

Mass spectrometry analysis: Data processing. A compilation of in-house software was used to convert mass spectrometric data (Raw file) to a mzXML format, as well as to correct monoisotopic m/z measurements and erroneous peptide charge state assignments. Assignment of MS/MS spectra was performed using the Sequest algorithm by searching the data against a protein sequence database including all entries the Mouse Uniprot database (download date June, 2014) containing known contaminants such as human keratins and its reverse decoy components (Elias and Gygi, 2007). Sequest searches were performed using a 20 ppm precursor ion tolerance and requiring each peptides N-/C- termini to have trypsin protease specificity, while allowing up to three missed cleavages. TMT tags on peptide N termini/lysine residues (+229.162932 Da) and carbamidomethylation of cysteine residues (+57.02146 Da) were set as static modifications while methionine oxidation (+15.99492 Da) was set as variable modification. A MS2 spectra assignment false discovery rate (FDR) of less than 1% was achieved by applying the target-decoy database search strategy (Elias and Gygi, 2007). Filtering was performed using an in-house linear discrimination analysis algorithm to create one combined filter parameter from the following peptide ion and MS2 spectra metrics: Sequest parameters XCorr and ΔCn, peptide ion mass accuracy and charge state, peptide length and mis-cleavages. Linear discrimination scores were used to assign probabilities to each MS2 spectrum for being assigned correctly and these probabilities were further used to filter the dataset to a 1% protein-level false discovery rate (Huttlin et al., 2010).

TMT reporter ion and quantitative data analysis. For quantification, a 0.003 m/z window centered on the theoretical m/z value of each ten reporter ions and the closest signal intensity from the theoretical m/z value was recorded. Reporter ion intensities were further de-normalized based on their ion accumulation time for each MS3 spectrum and adjusted based on the overlap of isotopic envelopes of all reporter ions (manufacturer specifications). Total signal to noise values for all peptides were summed for each TMT channel, and all values were adjusted to account for variance in sample handling. For each peptide, a total minimum signal to noise value of 200 was required (McAlister et al., 2012, Ting et al., 2011).

#### Pathway Enrichment Analysis (Proteomics)

For pathway analysis of cold exposure iBAT proteomics, proteins that were upregulated more than 1.5 fold compared to thermoneutrality (29°C) by *t* test (p < 0.05) were submitted to Enrichr for analysis (Kuleshov et al., 2016). Reactome pathways from Enrichr were presented in bubble chart format to show temporal changes. The Enrichr adjusted p value was used to determine significance. Significantly enriched Reactome pathways were manually selected.

#### Lipidomics

For targeted, quantitative lipidomics of cold exposed adipose tissue, mouse iBAT and scWAT of approximately 20 mg was added to 600 μL of 10 times diluted PBS in a 2mL tube from the Precelleys Lysing kit (KT03961-1-001.2). The samples were homogenized for 1 min (3X20 seconds with 10 seconds interval) at 4°C by using Precelleys Evolution homogenizer (Bertin Corp, MD). An aliquot of 25 μL was pipetted to determine the protein content (BCA protein assay kit, Thermo Scientific, Rockford, IL). The rest of homogenate was accurately transferred into a disposable glass culture test tube, and a mixture of lipid internal standards was added prior to lipid extraction for quantification of all reported lipid species. Lipid extraction was performed by using a modified Bligh and Dyer procedure as described previously (Wang and Han, 2014). Each lipid extract was resuspended into a volume of 200 μL of chloroform/methanol (1:1, v/v) per mg of protein and flushed with nitrogen, capped, and stored at −20°C for lipid analysis. For ESI direct infusion analysis, lipid extract was further diluted to a final concentration of ∼500 fmol/μL, and the mass spectrometric analysis was performed on a QqQ mass spectrometer (Thermo TSQ QUANTIVA, San Jose, CA) and Orbitrap mass spectrometer (Thermo LTQ Velos, San Jose, CA) equipped with an automated nanospray device (TriVersa NanoMate, Advion Bioscience Ltd., Ithaca, NY). Lipidomics data was corrected for protein content in each sample and is presented as absolute quantities within each lipid class in supplemental materials. Lipid internal standards are listed below (standards were purchased from Avanti Polar Lipids, except as noted):

1,2-Dimyristoleoyl-sn-glycero-3-phosphocholine (di14:1 PC)

1,2-Dipalmitoleoyl-sn-glycero-3-phosphoethanolamine (di16:1 PE)

1,2-Dipentadecanoyl-sn-glycero-3-phosphoglycerol (sodium salt) (di15:0 PG)

1,2-Dimyristoyl-*sn*-glycero-3-phospho-L-serine (sodium salt) (di14:0 PS)

1,2-Dimyristoyl-*sn*-glycero-3-phosphate (sodium salt) (di14:0 PA)

1,1’,2,2’-Tetramyristoyl cardiolipin (T14:0 CL)

7,7,8,8-d4-Palmitic acid (d4-16:0 NEFA) (Cambridge Isotope Laboratories)

N-Lauroryl sphingomyelin (N12:0 SM)

N-Heptadecanoyl ceramide (N17:0 Cer)

1-Heptadecanoyl-2-hydroxy-*sn*-glycero-3-phosphocholine (17:0 lysoPC)

1,2,3,4-13C4-Palmitoyl-L-carnitine hydrochloride (13C4-16:0 CN) (Sigma-Aldrich)

Triheptadecenoin (T17:1 TAG) (Nu Chek)

1,3- Dipentadecanoin (di15:0 DAG) (Nu Chek)

Monoheptadecenoin (17:1 MAG) (Nu Chek)

#### Tissue Cardiolipin Profiling by LC/MS

For cardiolipin profiling of adipose tissue, frozen tissue samples from 12 week old female mice were extracted in chloroform:methanol (2:1). Extraction volumes were adjusted according to wet tissue weight (20 mg tissue/400 μl extraction solvent) and internal standard 400 pmol cardiolipin C14 was added. Samples were sonicated, vortexed thoroughly and centrifuged (2,400 g, 2 min) before supernatants were transferred to new eppendorf tubes and washed with 0.2 volumes of water by thorough vortexing. Finally, the samples were centrifuged (400 g, 2 min) whereafter the lower organic phases were transferred to new tubes prior to lyophilization.

Samples were resuspended in 30 μl of solvent A (5:1:4 isopropanol/ methanol/water with 5 mM ammonium acetate and 0.1% acetic acid) before injection of 5 μl on an Agilent 1290 Infinity HPLC system equipped with an Eclipse Plus C18 RRHD column (2.1 x 50 mm, 1.8 μm) with a 50 mm guard-column, both kept at 45°C. The chromatographic gradient was run at flow rate of 350 μl/min and the following solvent composition of solvent A and B (99:1 isopropanol/water with 5 mM ammonium acetate and 0.1% acetic acid): 100% A from 0-3 min, 100-80% from 3-5 min, 80-70% from 5-25 min, 70-5% from 25-35 min, 5-5% from 35-36 min and 5-100% from 36-38 min before equilibration for 2 min with the initial conditions. Samples were analyzed with the MS operation in negative mode. All samples were run in randomized order.

The LC flow was coupled to an Agilent 6530 quadrupole time of flight (Q-TOF) mass spectrometer scanning from 70-1700 m/z with same settings as above. A library of the different cardiolipin species with retention times (RT) was constructed using Agilent MassHunter PCDL Manager. The identification of each compound was based on the exact mass. Chromatograms for all compounds were extracted and quantified using Agilent Profinder using a mass tolerance of 20 ppm and a retention time tolerance of 0.1 min.

#### Lipid Quantification from Cell Lysates by Thin Layer Chromatography

Primary brown adipocytes from Rosa26ERT2-*Cre*/*Crls1*^*f/f*^ mice were treated with tamoxifen (as described above in Tamoxifen-Induced Crls1 Knockout in Primary Adipocytes) and differentiated. On day 6 of differentiation, cells were incubated in differentiation media containing a mixture of 30 μM oleate, 15 μM palmitate, and 5 μM linoleate plus 0.5 μCi of [1- ^14^ C] linoleate for 6 h. Total lipids were extracted from the cells, and neutral lipids were separated from phospholipids by thin layer chromatography (heptane:isopropyl ether:acetic acid; 60:40:4; v/v/v). Phospholipids were extracted from the silica gel and different classes were separated using chloroform:ethanol:water:triethalamine (30:35:7:35; v/v/v/v) (Vaden et al., 2005). Radioactivity in each class was measured using an AR-2000 radio-TLC imaging scanner (Eckert & Zeigler, Berlin, Germany).

#### Gene Expression Analysis (RT qPCR and RNA-seq)

For gene expression analysis of tissue and cell samples, RNA extraction was completed using TRI reagent (Sigma-Aldrich). cDNA synthesis was carried out using a High Capacity cDNA Reverse Transcription Kit (Applied Biosystems). Expression data was analyzed with the ΔΔC_T_ method. Gene expression data was normalized to *36b4* (mouse and cell lines) or *PPIA* (human tissue). All primers are listed in Table S2.

For generation of RNA-seq libraries, polyadenylated mRNA was isolated from 1 μg of total RNA by incubation with oligo-dT beads and prepared according to the manufacturer’s instructions (TruSeq 2, Illumina). Samples were sequenced on the Illumina HiSeq 1500 platform. Sequencing reads were mapped to the mouse reference genome (version mm9) using STAR (Dobin et al., 2013). Tag directories were generated using HOMER (Heinz et al., 2010) and exon reads were counted using iRNA-seq (Madsen et al., 2015). Normalization and identification of differentially expressed genes was performed using DESeq2 (Love et al., 2014). For calling differentially expressed genes, a FDR < 0.01 and threshold of minimum 10 reads per kb on average for the given condition were used. For analysis of RNA-seq pathway enrichment in AdCKO iBAT, the list of genes downregulated by a third or more with a p value < 1x10^-5^ were submitted to Enrichr for analysis (Kuleshov et al., 2016). Reactome pathways from Enrichr were manually selected for presentation. RNA-seq data was deposited in GEO (GEO: GSE110297).

#### Metabolomics

Frozen tissue samples from 12 week old female mice was extracted in methanol:acetonitrile:water (5:3:2) including internal standards (0.5 μM butyrylcarnitine D3, 0.25 μM hexadecanoyl D3 and 5 μM heavy labeled amino acid mix standard). Extraction volumes were adjusted according to wet tissue weight (20 mg tissue/200 μl extraction solvent). Samples were sonicated, vortexed thoroughly and centrifuged (21,100 g at 4°C) for 10 min. Subsequently, 50 μl of the supernatants were transferred into new tubes before lyophilization.

Metabolites were profiled using LC-MS with RP separation. Samples were redissolved in 20 μl 1% formic acid (FA) in water before injection of 8 μl on a Agilent 1290 Ininity HPLC system (Agilent Technologies, Santa Clara, CA) equipped with an Agilent Zorbax Eclipse Plus C18 column (2.1 x 150 mm, 1.8 μm) with a 50 mm guard-column, both kept at 40°C. The chromatographic gradient was run at a flow rate of 300 μl/min with the following solvent composition of A (0.1 % FA, water) and B (0.1% FA, acetonitrile): 97% A from 0-5 min, 97-85% A from 5-8 min and 85-60% A from 8-18 min before equilibration for 3 min with the initial conditions. Samples were analyzed twice with the MS operating in both positive and negative ion mode, respectively.

All samples were run in randomized order and one mastermix sample (equal amounts pooled from all samples) was included for all setups and run in all-ion fragmentation mode with collision energy of 20 V in order to produce fragments for identification of the metabolites. The LC flow was coupled to an Agilent 6530 quadrupole time of flight (Q-TOF) mass spectrometer scanning from 70-1050 m/z. Libraries of metabolites with retention time (RT) were constructed using Agilent MassHunter PCDL Manager. The identification of each compound was based on exact mass, RT of synthetic standards and/or comparison of fragments with the Metlin MS/MS database (https://metlin.scripps.edu/). Chromatograms for all compounds were extracted and quantified using Agilent Profinder using a mass tolerance of 20 ppm and a retention time tolerance of 0.1 min.

#### CRLS1 Immunoblotting (Western)

Tissue samples were homogenized with metal beads using a TissueLyser (Quigen) with 1.5 minutes of lysis at a frequency of 20s^-1^ in 1x RIPA buffer (Millipore) buffer supplemented with Pierce Protease and Phosphatase Inhibitor Mini Tablets (Thermo Scientific). Samples were prepared with sample buffer (NuPage) and DTT, boiled and analyzed. Proteins were run on 3-8% SDS-PAGE gels (NuPage) and transferred to a PVDF membrane using a semi-dry blotting technique. Custom rabbit polyclonal anti-CRLS1 antibodies were ordered from Genscript (PolyExpress Gold Package; SC1649). Antibodies were separately produced against three CRLS1 peptide antigens (RPPGARLGRGGSRRC, CSGAGKAAPEPAAGG and SARWVPASAASSYEC). Amersham ECL Rabbit IgG, HRP-linked whole Ab (from donkey) was used as the secondary antibody (GE Healthcare Life Sciences NA934).

#### Gain-of-Function in Primary Mouse Adipocytes

A mouse *Crls1* (NM_001024385) cDNA ORF clone in pcDNA3.1+/C-(K)DYK vector was synthesized by Genscript (OMu17939). One million primary brown or beige-like adipocytes were electroporated on differentiation day 3 with sterile plasmid using a Nucleofector II device with Cell Line Nucleofector Kit L (Lonza VACA-1005) and protocol A-033. Electroporated cells were plated onto either a 96-well plate for gene expression analysis (45k cells/well) or a 96-well Seahorse plate for respirometry analysis (30k cells/well). Cells were harvested or assayed four days after electroporation.

#### Gain-of-Function in Human Adipocytes via CRISPRa SAM

Lentivirus expressing dCasp-VP64 and MS2-P65-HSF1 were generated in HEK-293 cells by co-transfecting with psPAX.2 and psMD2.g plasmids using Superfect transfection reagent (Qiagen). Virus-containing supernatant was harvested 72 hours post transfection and concentrated using Lenti-X concentrator (Clontech). Immortalized human white adipose progenitor cells derived from human neck fat (see Human Adipocyte Isolation, Immortalization and Culture above) were transduced with virus and 72 hours later treated with Blasticidin (final concentration of 5 ug/ml) and Hygromycin (final concentration of 25ug/ml). Expression of dCasp-VP64 and MS2-P65-HSF1 were confirmed by qPCR. The *CRLS1* sgRNA was designed to target the *CRLS1* promoter at the -101 position upstream from the transcription start site with the following sequence: 5’-CATCAGGCTCAGTGGGTTTT-3’. The sgRNA was cloned into the gRNA lentiviral backbone. Lentivirus production, up-concentration and human progenitor transduction were carried out as described for the other lentiviral constructs. An empty vector (EV) lentiviral backbone-expressing cell line was established to control for lentiviral infection and drug selection. The sgRNA was transduced into cells stably expressing the core components of the CRISPRa-SAM system: dCAS9-VP64 and MS2-P65-HSF1 to drive expression of the *CRLS1* gene by activating the endogenous promoter. Selection of sgRNA-expressing cells was done using Zeocin (final concentration of 50 μg/ml). Zeocin was kept in media during passaging of the cell lines. Confirmation of *CRLS1* overexpression (OE) was done in progenitor cells using qPCR. For oxygen consumption rate experiments, *CRLS1* OE and EV cells were differentiated as previously described (Xue et al., 2015).

#### Loss-of-Function in Mouse Adipocytes with siRNA

At approximately 80% confluency, 160k preadipocytes were reverse transfected onto 12-well plates containing 50 pmoles/well of siRNA targeting *Crls1* (Sigma, SASI_Mm01_00094096) or control siRNA (Sigma, SIC001) using RNAiMAX (Thermo Scientific, 13778100). After reaching confluency, cells were differentiated as described above. On day 3 of differentiation, cells were again reverse transfected onto either a 96-well plate for gene expression analysis (60k cells/well; 10 pmoles of siRNA) or a 96-well Seahorse plate for respirometry analysis (30k cells/well; 5 pmoles of siRNA). Cells were harvested or assayed on differentiation day 7. For experiments with both Crls1 and *Chop10* (*Ddit3,* Sigma, SASI_Mm01_00085431) knockdown, cells were treated with siRNA against *Crls1* as described above, and treated with *Chop10* siRNA alone or with *Crls1* siRNA on day 3.

#### Tamoxifen-Induced *Crls1* Knockout in Primary Adipocytes

Primary preadipocytes were isolated as described in Primary Mouse Adipocyte Cultures above. Three days after seeding, primary preadipocytes were approximately 50% confluent and treated with 1 μM 4-hydroxytamoxifen (Sigma, H6278). This treatment was repeated 24 hours later. Once cells were confluent, they were differentiated as detailed above. Cells were replated onto either a 96-well plate for gene expression analysis (60k cells/well) or a 96-well Seahorse plate for respirometry analysis (30k cells/well). Cells were harvested or assayed on differentiation day 7.

#### Mitochondrial Isolation

Mitochondria were isolated by differential centrifugation. Freshly dissected interscapular and sub-scapular iBAT was minced with scissors in ice cold isolation buffer (IB, 250 mM sucrose in water). Minced tissue was homogenized in 20 ml of IB using a Potter-Elvehjem type grinder at 500 rpm for 2 min (Velp Scientific DLS). The homogenate was filtered with a cotton cloth and centrifuged at 10,000 x g for 10 min at 4°C. The supernatant was discarded, fat on the tube wall was carefully removed with paper tissue, and the pellet was resuspended in 2 ml of IB. The suspension was centrifuged again at 800 x g for 10 min at 4°C. The supernatant was transferred to a new tube and centrifuged again at 10,000 x g for 10 min at 4°C. The final pellet was frozen at -80°C for storage.

#### Cellular and Mitochondrial Bioenergetics

Primary mouse adipocytes were seeded onto 96-well Seahorse plates on day 2 of differentiation at 20,000 cells per well. On day 7 of differentiation, cells were rinsed once and media was replaced with 165 μl basal DMEM (Sigma Aldrich D5030) containing 25 mM glucose, 1 mM sodium pyruvate and 0.5% BSA (Sigma Aldrich A7030). After 1 hour in a non-CO_2_ incubator at 37°C, plates were inserted into a Seahorse XF96 Analyzer. After 3 basal measurements, oligomycin was injected for a final concentration of 1μM to inhibit ATP synthesis. Next, NE was injected in order to measure adrenergic-induced uncoupled respiration (1 μM for brown adipocytes, 5 μM for beige-like adipocytes). After NE-induced uncoupled respiration was determined, FCCP was injected (final concentration 1 μM) to determine maximal respiratory capacity. Finally, a mixture of rotenone and antimycin was injected to inhibit all mitochondrial respiration (1 μM each). NE-induced uncoupled respiration curves were calculated by subtracting the oligomycin-dependent OCR from the OCR values after NE treatment. Cumulative NE-induced uncoupled respiration was calculated as the area under curve. Metabolic parameters for basal mitochondrial, coupled, NE-induced uncoupled and maximum respiration were calculated relative to baseline after subtracting non-mitochondrial OCR values (OCR after rotenone and antimycin). Immortal mouse brown adipocytes were assessed similarly. Bioenergetics of immortal human adipocytes was assessed similarly, except preadipocytes were seeded directly onto 96-well Seahorse plates and differentiated in-well without rosiglitazone, no BSA was used in the assay media and adrenergic-induced uncoupled respiration was determined by treatment with 10 μM forskolin instead of NE. For mitochondrial bioenergetics, mitochondria pellets were resuspended in assay buffer (125 mM sucrose, 20 mM K+-Tes (pH 7.2), 2 mM MgCl_2_, 1 mM EDTA, 4 mM KH2PO4, and 0.1% fatty-acid-free BSA), and then 2 μg of isolated mitochondria (as measured by protein content) was added to each well of a 96-well Seahorse plate. The plate was spun at 2,000 x g for 20 minutes at 4°C. After 3 basal readings, 5 mM glycerol-3-phosphate (G3P) was injected to each well, followed by 3 measurements and then 3 mM guanosine diphosphate (GDP) was added to inhibit UCP1.

#### Quantification of Mitochondrial Mass (Mitochondrial DNA)

DNA was isolated from adipose tissue using a Gene Elute Mammalian DNA Miniprep Kit (Sigma #G1N70). The ratio of mitochondrial DNA (mtDNA) to genomic DNA was determined by performing qPCR. Four individual primer sets were used for mitochondrial DNA that target the mitochondrial genes *Nd1*, *Nd2*, *Co2* and *Cytb* (Table S2). Two pairs of primers were used to amplify nuclear DNA from within the *Ucp1* and *Pparg* loci (Table S2). All primer pairs were run in individual reactions. The mtDNA/nucDNA was calculated separately for each primer pair using 2^∗^2ˆ(Ct_nucDNA_-Ct_mtDNA_). The final mtDNA/nucDNA ratio for each sample was calculated by averaging the ratio obtained from each primer pair. Expression data was analyzed with the ΔΔC_T_ method. All primers are listed in Table S2.

#### Quantification of Mitochondrial Mass (Citrate Synthase Activity)

Previously frozen in liquid nitrogen brown adipose tissue (5-10 mg) was homogenized in 500 μl lysis buffer (containing Glycerol 10 %, IGEPAL 1%, NaCl 150 mM, HEPES 50 mM, β-glycerophosphate 20 mM, NaF 10 mM, EDTA 1 mM, EGTA 1 mM, Na-Butyrate 1 mM, Na-pyrophosphate 20 mM, Na_3_VO_4_ 2 mM, Thiamet G 4 μM, Nicotinamide 5 μM, 1x cOmplete Mini Roche protease inhibitor cocktail using QIAGEN TissueLyser II bead homogenizer. Tissue homogenates were rotated end-over-end at 4°C for 1 hour, followed by centrifugation at 16,000 g for 20 minutes at 4°C and supernatants were used for the following measurements. Citrate synthase activity was measured spectrometrically (CLARIOstar microplate reader, BMG LABTECH) in diluted tissue extracts (5-20 times serial dilutions in 0.1 M Tris-HCl pH 8.1) at 37°C in a solution containing 500 μM oxaloacetate, 100 μM DTNB (5,5-dithio-bis-(2-nitrobenzoic acid), 400 μM Acetyl-CoA, 0.1 M Tris-HCl (pH 8.1) based on the absorption at 412 nm by the product thionitrobenzoic acid (TNB). Citrate synthase activity was calculated using Equation 1 and expressed as nanomoles of substrate per minute per microgram of protein. Protein concentration was measured in the diluted tissue extracts using Precise BCA Protein Assay Kit (Thermo Scientific).

(Equation 1) $$\mathrm{citrate}\;\mathrm{synthase}\;\mathrm{activity}=\frac{\Delta A_{412}/\min\cdot V\left(mL\right)}{\varepsilon^{mM}\cdot L\left(cm\right)\cdot \mathrm{total protein}\;\left(g\right)}$$

$\varepsilon^{mM}=13.6\;mM^{-1}\;cm^{-1}$ (the extinction coefficient of TNB at 412 nm), L = 0.535 cm

#### BN-PAGE and Native Immunoblotting of Mitochondrial Complexes and Supercomplexes

Analysis of mitochondrial complexes and supercomplexes was performed similarly as previously described (Jha et al., 2016). Frozen mitochondrial isolates were removed from -80°C storage and resuspended in either 250 μl (for control), or 100 μl (for AdCKO) of ice cold IB. Protein content was measured with a micro BCA Protein Assay Kit (Thermo Scientific). Fifty μg of mitochondrial protein was aliquoted into 1.5 ml tubes and centrifuged at 7,000 x g for 10 min at 4°C. The supernatant was discarded and the pellet was either used immediately or stored at -80°C for BN-PAGE. Fifty μg of mitochondrial protein was resuspended in 20 μl of sample buffer cocktail, containing 5 μl of 4x NativePAGE sample buffer, 8 μl of 5% digitonin and 7 μl of water. After solubilization by gently pipetting up and down, samples were incubated for 20 min on ice, then centrifuged at 20,000 x g for 10 min at 4°C and supernatants were transferred to a new tube. Next, 2 μl of G-250 sample additive was added to each sample and they were loaded into a NativePAGE 3-12% gradient gel. Fifteen μl of Native Mark Unstained Protein Standard (Invitrogen LC0725) was also loaded into the gel. The outer chamber of the running apparatus was filled with 1x Native PAGE running buffer, while the inner chamber of the gel apparatus was filled with 1x Native PAGE running buffer that contained 0.02% Coomassie Brilliant Blue G-250 (weight/vol). After 30 min at 150 V, the buffer in the inner chamber was replaced with Native PAGE running buffer containing 0.002% Coomassie Brilliant Blue G-250 (weight/vol). The gel was then run an additional 60 min at 250 V. The gel was then washed with water and imaged. Next, the gel was incubated in 1x NuPAGE Transfer Buffer for 15 minutes with shaking. The proteins were transferred to a PVDF membrane using a Power Blotter (Thermo Scientific) set to 25 volts and 1 amp, and run for 60 minutes. Following transfer, the membrane was placed in 8% acetic acid for 5 min with shaking and air-dried. For immunoblotting, the membrane was incubated in methanol 3 times for 5 min with shaking. Next, it was incubated in blocking solution (Western Breeze Chromogenic Immunodetection System, Novex # WB7103) for 30 min, then washed 2 times with water for 5 min and incubated with primary antibody solution for 90 minutes. Primary antibody solution consisted of 2 ml of Blocker/Diluent part A, 1 ml of Blocker/Diluent part B (Western Breeze Chromogenic Immunodetection System), 7 ml water, 10 μl of anti-OxPhos antibody cocktail (Life technologies, cat. no. 457999), and 5 μl of anti-MTCO1 antibody (Abcam, cat. no. ab14705). The membrane was then washed with 1x antibody wash solution 2 times 5 min (Western Breeze Chromogenic Immunodetection System), followed by 2 times 5 min washes in water. The membrane was then incubated with secondary antibody solution (Western Breeze Chromogenic Immunodetection System) for 45 minutes with gentle shaking. The membrane was then washed with 1x antibody wash solution 2 times 5 min (Western Breeze Chromogenic Immunodetection System), followed by 2 times 5 min washes in water. Finally, the membrane was incubated with chromogenic substrate (Western Breeze Chromogenic Immunodetection System) until bands were clear and washed twice with water before being air-dried, scanned and bands quantified using a ChemiDoc XRS+ and Image Lab 6.0 software (Bio-Rad). Protein band quantities were normalized to total Coomassie stained volume per lane.

#### Quantification of Intracellular Triglycerides

In order to determine tissue triglyceride content, lipids were extracted from 50-200 mg of tissue by overnight incubation at 55°C in ethanolic KOH (66.6% ethanol with 33.3% 30% KOH). Samples were brought to a volume of 1200 μl with 50% ethanol then spun for 5 minutes at maximum speed in a microcentrifuge. After mixing 100 μl of supernatant with 100 μl of 0.5 M MgCl_2_, samples were kept on ice for 10 minutes. They were then centrifuged for 5 minutes at maximum speed in a microcentrifuge and supernatant was moved to a new tube. Glycerol standards were prepared ranging from 1,000 mg/dl down to 1 mg/dl. In a standard 96-well plate, 3 μl of sample or standard was mixed with 300 μl of Infinity Triglyceride Liquid Stable Reagent (#TR22421, Thermo Fisher Scientific) and incubated at 37°C for 10 minutes before reading. The unknown triolein equivalents (TE) were interpolated from the standard curve using a least squares (ordinary) fit line calculated with Prism Graphpad software. The total liver triglycerides per sample was calculated in mg/g tissue by TE^∗^2^∗^0.012/(tissue weight in grams).

#### Indirect Calorimetry

Indirect calorimetry was performed using Home Cage System Phenomaster (TSE Systems). Briefly, animals were acclimated in training cages for 3-5 days prior to the measurement and were allowed 24 hours to acclimate to the TSE cabinets. Gas exchanges and food intake were recorded every 5 minutes. Temperature and activity monitoring was conducted using implanted E-Mitter as described below. All metabolic phenotyping data was analyzed by averaging data from two days of measurements.

#### Telemetric Temperature and Activity Monitoring

For implantation of telemetric temperature and activity monitoring devices, mice were anesthetized with isofluorane and kept on heated pads during the procedure. G2 E-Mitter Telemetry System devices (Starr Life Sciences) were surgically implanted subcutaneously directly over the interscapular brown adipose depots and sutured into place. ER4000 Receivers were placed under the cages within the TSE cabinets. Temperature and physical activity data was integrated into the Phenomaster software.

#### Cold Tolerance

Mice were pair-housed at 22°C. Food was withdrawn at the commencement of the cold tolerance test as animals were transferred to new cold acclimated cages containing only bedding material. The cages were then placed into climate controlled rodent incubators (Memmert HPP750Life) set to 5°C. Core body temperature was obtained with a Homeothermic Monitor (Harvard Apparatus) by gently inserting a thermal probe into the mouse rectum.

#### Brown Adipose Thermogenic Capacity Procedure

Oxygen consumption and iBAT temperature were measured using the Phenomaster system as described above with implanted telemetric E-Mitter devices. Male mice at 16 weeks of age were anaesthetized with Pentobarbital (75mg/kg). After oxygen consumption measurement stabilized again in the system, 1mg/kg of NE was administered subcutaneously and the measurement was continued until homeostasis was reached. All data was collected every 3 minutes. The climate chamber was kept at 33°C during the procedure.

#### Small Animal FDG PET/CT Imaging

All imaging experiments were performed with an approved protocol from the University of Copenhagen, project number P15-352. Twenty week old female mice were housed at thermo neutrality the night before imaging and fasted from 7 AM on the day of imaging. FDG was administered intraperitoneally between 10AM–12PM. The average radioactive dose was 7.7 MBq (range: 6.5-9.1 MBq). CL 316,243 (1 mg/kg) was administered subcutaneously 15 min prior to FDG administration. Small animal PET/CT (Inveon Multimodality PET/CT scanner; Siemens) was performed 1 hour after FDG administration. Mice were anaesthetized by sevoflurane 30 minutes after tracer injection until the end of the imaging session. Heating was applied in order to maintain normal body temperature. PET data were acquired in list mode for 300s, and images were reconstructed using a 3-dimensional maximum a posteriori algorithm with CT-based attenuation correction. CT images were acquired using 360 projections, 65 kV, 500 mA, and 400 ms exposure and reconstructed with an isotropic voxel size of 0.210 mm. Images were analyzed using the Inveon software (Siemens). Quantitative analysis of the FDG uptake was performed by manually drawing region of interests over the areas containing iBAT based on the CT images. The FDG uptake was expressed as % injected dose per gram tissue (%ID/g).

#### Sample Preparation for Electron Microscopy

Ten-week old female mice were anesthetized with isofluorane and perfused with 2% glutaraldehyde. iBAT samples were collected and fixed with 2% v/v glutaraldehyde in 0.05 M sodium phosphate buffer (pH 7.4) for 48 h followed by three times rinse in 0.15 M sodium cacodylate buffer (pH 7.4). The specimens were subsequently postfixed in 1% w/v OsO4 and 0.05M potassium ferricyanide in 0.12 M sodium cacodylate buffer (pH 7.4) for 2 h. Followed by én bloc stain in 1% uranyl acetate in distilled water over night, the specimens were dehydrated in graded series of ethanol, transferred to propylene oxide and embedded in Epon according to standard procedures.

#### TEM

Sections, approximately 80 nm thick, were cut with a Leica UC7 microtome and collected on copper grids with Formvar supporting membranes. Sections were stained with uranyl acetate and lead citrate and subsequently examined with a Philips CM 100 TEM (Philips, Eindhoven, The Netherlands), operated at an accelerating voltage of 80 kV. Digital images were recorded with an OSIS Veleta digital slow scan 2k x 2k CCD camera and the ITEM software package.

#### FIB/SEM

The Epon embedded specimens were analyzed in Quanta FEG 3D FIB-SEM (FEI, The Netherlands) equipped with a gallium ion source for milling and a dedicated backscattered electron detector for imaging (vCD). The surface of the block and the trimmed edge was located with the secondary electron detector in standard SEM mode. Definition of an area of interest was acquired with the vCD detector at 20 kV accelerating tension. For focused ion beam milling the block was tilted to 52° and the edge of the block aligned at eucentric height, followed by crossover alignment of both electron and ion beams. The ion beam was used in conjunction with a gas injection system to deposit a 1 μm layer of platinum above the region of interest to reduce milling artifacts. Next, trenches approximately 20 μm wide were milled at high beam current on both sides of the region of interest to avoid deposition artifacts. The G2 Slice and View software (FEI) was used for automatic milling (50 nm) and image recording with automatic refocusing of the exposed surface.

#### SEM 3D Image Reconstruction

Two digital image dataset of 562 and 579 images respectively, were recorded from control and AdCKO sample blocks. The images with the specifications of 2048 x 1768 pixels in 8 bits were assembled and automatically aligned in Amira 6.0.0 – ResolveRT. Voxel size was 33.5 x 42.3 x 50 nm. From the two image stacks, blood vessels were automatically masked using the LabelField and were included for 3D reconstruction. 3D models were generated by SurfaceGen and projected by SurfaceView. The lipid droplets and cellular organelles were visualized using the Volren volume-rendering tool. Finally, the images were captured in high resolution and the cellular mitochondrial (and other small organelles) were manually false coloured green in Photoshop CC. All images were post processed in Photoshop CC.

#### Body Composition Analysis

Mouse fat and lean mass was measured using nuclear magnetic resonance technology with an EchoMRI 4 in 1 Body Composition Analyzer.

#### Mouse Metabolic and HFD Studies

For glucose and insulin tolerance tests, mice were fasted for 4 hours starting at 9:00. For glucose challenge, mice were given an intraperitoneal injection of 2 g/kg body weight glucose. For ITT, mice were injected with 0.75 units/kg body weight (chow-fed) or 1.5 units/kg body weight (HFD-fed) of insulin (Novo Nordisk, Humulin R). Glucose was measured with Contour XT glucometers (Bayer). GTT and ITT tests were performed 1 week apart. At 16 weeks of age, male mice were kept at 22°C and given ad libitum access to a high fat diet containing 60% kcal from fat, 20% kcal from carbohydrate and 20% kcal from protein (Research Diets, D12492). Body weights were recorded weekly.

### Quantification and Statistical Analysis

Unless otherwise noted, statistical analysis was performed with GraphPad Prism 7.0. Statistical parameters, including the value of n, are noted in figure legends. Unless otherwise noted, all data are presented as means ± SEM. Statistical analysis of data with more than 2 groups and without repeated measures was with one-way ANOVA. Repeated measures data with more than 2 groups was assessed with two-way repeated measures (RM) ANOVA. Data with 2 groups was analyzed by two-tailed Student’s *t* test. Correction for multiple comparisons was done using the Holm-Sidak method. Proteomics data was statistically analyzed with Perseus software (http://www.perseus-framework.org) using ANOVA with a permutation-based false discovery rate with 250 randomizations. Heatmaps were prepared with Metaboanalyst software (http://www.metaboanalyst.ca/) and GraphPad Prism.

### Data and Software Availability

The accession number for the iBAT RNA-seq data is GEO: GSE110297.

## Supplemental Information

Document S1. Figures S1–S6 and Tables S1 and S2

Video S1. Video of Focused Ion Beam Scanning Electron Microscopy Image Stack through 26 μm of Control BAT at 5,000× Magnification, Related to Figures 4 and S5

Video S2. Video of Focused Ion Beam Scanning Electron Microscopy Image Stack through 26 μm of AdCKO BAT at 5,000× Magnification, Related to Figures 4 and S5

Video S3. Video of FDG-PET/CT Imaging of CL-316,243-Induced Glucose Uptake in a Representative Control Mouse, Related to Figure 4

Video S4. Video of FDG-PET/CT Imaging of CL-316,243-Induced Glucose Uptake in a Representative AdCKO Mouse, Related to Figure 4

Data S1. Proteomics Analysis of iBAT from Cold Exposed Mice, Related to Figure 1

Data S2. Lipidomic Analysis of iBAT and scWAT from Cold Exposed Mice, Related to Figure 2

Document S2. Article plus Supplemental Information
