# Supplementary material for: Cardiolipin Synthesis in Brown and Beige Fat Mitochondria Is Essential for Systemic Energy Homeostasis
Source: Cell Metab. 2018 Jul 3;28(1):159–174.e11. doi: 10.1016/j.cmet.2018.05.003 (PMC6038052; doi:10.1016/j.cmet.2018.05.003)
Supplement: Document S1. Figures S1–S6 and Tables S1 and S2 [file mmc1.pdf]

Cell Metabolism, Volume *28*

Supplemental Information

Cardiolipin Synthesis in Brown and Beige Fat Mitochondria Is Essential for Systemic Energy Homeostasis

Elahu G. Sustarsic, Tao Ma, Matthew D. Lynes, Michael Larsen, Iuliia Karavaeva, Jesper F. Havelund, Carsten H. Nielsen, Mark P. Jedrychowski, Marta Moreno-Torres, Morten Lundh, Kaja Plucinska, Naja Z. Jespersen, Trisha J. Grevengoed, Barbara Kramar, Julia Peics, Jakob B. Hansen, Farnaz Shamsi, Isabel Forss, Ditte Neess, Susanne Keipert, Jianing Wang, Katharina Stohlmann, Ivan Brandslund, Cramer Christensen, Marit E. Jørgensen, Allan Linneberg, Oluf Pedersen, Michael A. Kiebish, Klaus Qvortrup, Xianlin Han, Bente Klarlund Pedersen, Martin Jastroch, Susanne Mandrup, Andreas Kjær, Steven P. Gygi, Torben Hansen, Matthew P. Gillum, Niels Grarup, Brice Emanuelli, Søren Nielsen, Camilla Scheele, Yu-Hua Tseng, Nils J. Færgeman, and Zachary Gerhart-Hines

A

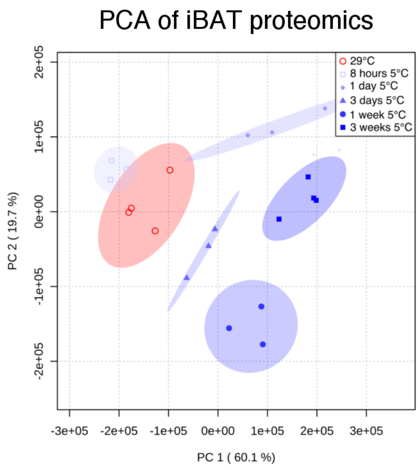

PCA of iBAT proteomics
29°C
8 hours 5°C
1 day 5°C
3 days 5°C
1 week 5°C
3 weeks 5°C
PC 2 (19.7 %)
PC 1 (60.1 %)
2e+05
1e+05
0e+00
-1e+05
-2e+05
-3e+05
-2e+05
-1e+05
0e+00
1e+05
2e+05
3e+05

B

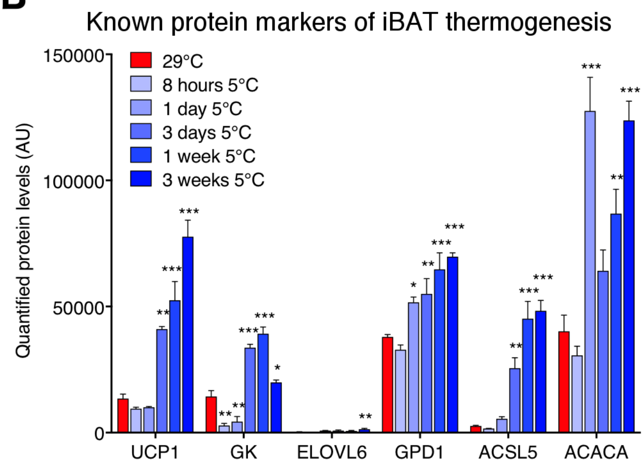

Known protein markers of iBAT thermogenesis
29°C
8 hours 5°C
1 day 5°C
3 days 5°C
1 week 5°C
3 weeks 5°C
Quantified protein levels (AU)
150000
100000
50000
0
UCP1
GK
ELOVL6
GPD1
ACSL5
ACACA

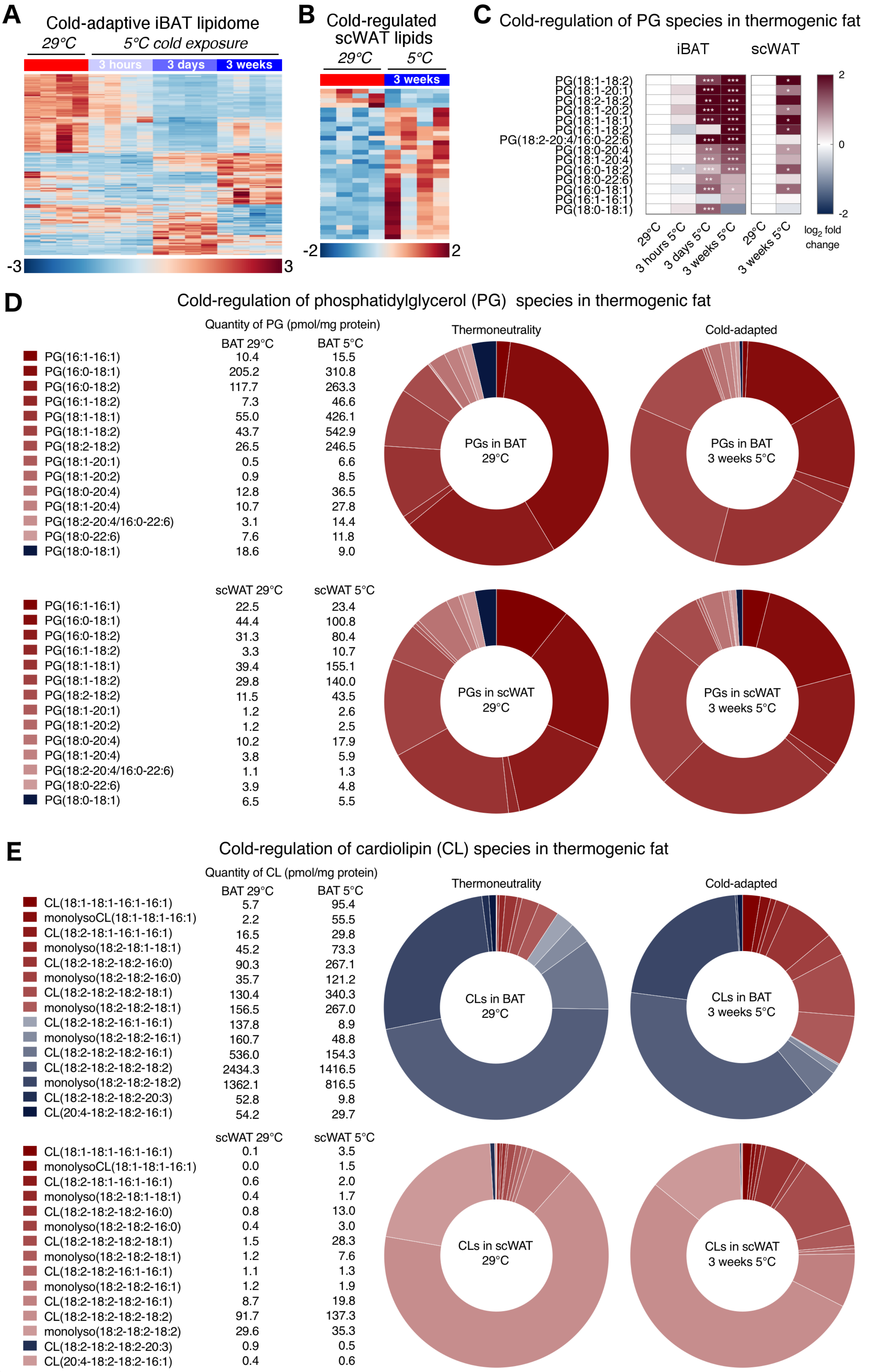
A
Cold-adaptive iBAT lipidome
29°C
5°C cold exposure
3 hours
3 days
3 weeks
-3
3
B
Cold-regulated scWAT lipids
29°C
5°C
3 weeks
-2
2
C
Cold-regulation of PG species in thermogenic fat
iBAT
scWAT
PG(18:1-18:2)
PG(18:1-20:1)
PG(18:2-18:2)
PG(18:1-20:2)
PG(18:1-18:1)
PG(16:1-18:2)
PG(18:2-20:4/16:0-22:6)
PG(18:0-20:4)
PG(18:1-20:4)
PG(16:0-18:2)
PG(18:0-22:6)
PG(16:0-18:1)
PG(16:1-16:1)
PG(18:0-18:1)
29°C
3 hours 5°C
3 days 5°C
3 weeks 5°C
29°C
3 weeks 5°C
2
0
-2
log2 fold change
D
Cold-regulation of phosphatidylglycerol (PG) species in thermogenic fat
Quantity of PG (pmol/mg protein)
BAT 29°C | BAT 5°C
PG(16:1-16:1) 10.4 15.5
PG(16:0-18:1) 205.2 310.8
PG(16:0-18:2) 117.7 263.3
PG(16:1-18:2) 7.3 46.6
PG(18:1-18:1) 55.0 426.1
PG(18:1-18:2) 43.7 542.9
PG(18:2-18:2) 26.5 246.5
PG(18:1-20:1) 0.5 6.6
PG(18:1-20:2) 0.9 8.5
PG(18:0-20:4) 12.8 36.5
PG(18:1-20:4) 10.7 27.8
PG(18:2-20:4/16:0-22:6) 3.1 14.4
PG(18:0-22:6) 7.6 11.8
PG(18:0-18:1) 18.6 9.0
Thermoneutrality
Cold-adapted
PGs in BAT 29°C
PGs in BAT 3 weeks 5°C
scWAT 29°C | scWAT 5°C
PG(16:1-16:1) 22.5 23.4
PG(16:0-18:1) 44.4 100.8
PG(16:0-18:2) 31.3 80.4
PG(16:1-18:2) 3.3 10.7
PG(18:1-18:1) 39.4 155.1
PG(18:1-18:2) 29.8 140.0
PG(18:2-18:2) 11.5 43.5
PG(18:1-20:1) 1.2 2.6
PG(18:1-20:2) 1.2 2.5
PG(18:0-20:4) 10.2 17.9
PG(18:1-20:4) 3.8 5.9
PG(18:2-20:4/16:0-22:6) 1.1 1.3
PG(18:0-22:6) 3.9 4.8
PG(18:0-18:1) 6.5 5.5
PGs in scWAT 29°C
PGs in scWAT 3 weeks 5°C
E
Cold-regulation of cardiolipin (CL) species in thermogenic fat
Quantity of CL (pmol/mg protein)
BAT 29°C | BAT 5°C
CL(18:1-18:1-16:1-16:1) 5.7 95.4
monolysoCL(18:1-18:1-16:1) 2.2 55.5
CL(18:2-18:1-16:1-16:1) 16.5 29.8
monolyso(18:2-18:1-18:1) 45.2 73.3
CL(18:2-18:2-18:2-16:0) 90.3 267.1
monolyso(18:2-18:2-16:0) 35.7 121.2
CL(18:2-18:2-18:2-18:1) 130.4 340.3
monolyso(18:2-18:2-18:1) 156.5 267.0
CL(18:2-18:2-16:1-16:1) 137.8 8.9
monolyso(18:2-18:2-16:1) 160.7 48.8
CL(18:2-18:2-18:2-16:1) 536.0 154.3
CL(18:2-18:2-18:2-18:2) 2434.3 1416.5
monolyso(18:2-18:2-18:2) 1362.1 816.5
CL(18:2-18:2-18:2-20:3) 52.8 9.8
CL(20:4-18:2-18:2-16:1) 54.2 29.7
Thermoneutrality
Cold-adapted
CLs in BAT 29°C
CLs in BAT 3 weeks 5°C
scWAT 29°C | scWAT 5°C
CL(18:1-18:1-16:1-16:1) 0.1 3.5
monolysoCL(18:1-18:1-16:1) 0.0 1.5
CL(18:2-18:1-16:1-16:1) 0.6 2.0
monolyso(18:2-18:1-18:1) 0.4 1.7
CL(18:2-18:2-18:2-16:0) 0.8 13.0
monolyso(18:2-18:2-16:0) 0.4 3.0
CL(18:2-18:2-18:2-18:1) 1.5 28.3
monolyso(18:2-18:2-18:1) 1.2 7.6
CL(18:2-18:2-16:1-16:1) 1.1 1.3
monolyso(18:2-18:2-16:1) 1.2 1.9
CL(18:2-18:2-18:2-16:1) 8.7 19.8
CL(18:2-18:2-18:2-18:2) 91.7 137.3
monolyso(18:2-18:2-18:2) 29.6 35.3
CL(18:2-18:2-18:2-20:3) 0.9 0.5
CL(20:4-18:2-18:2-16:1) 0.4 0.6
CLs in scWAT 29°C
CLs in scWAT 3 weeks 5°C

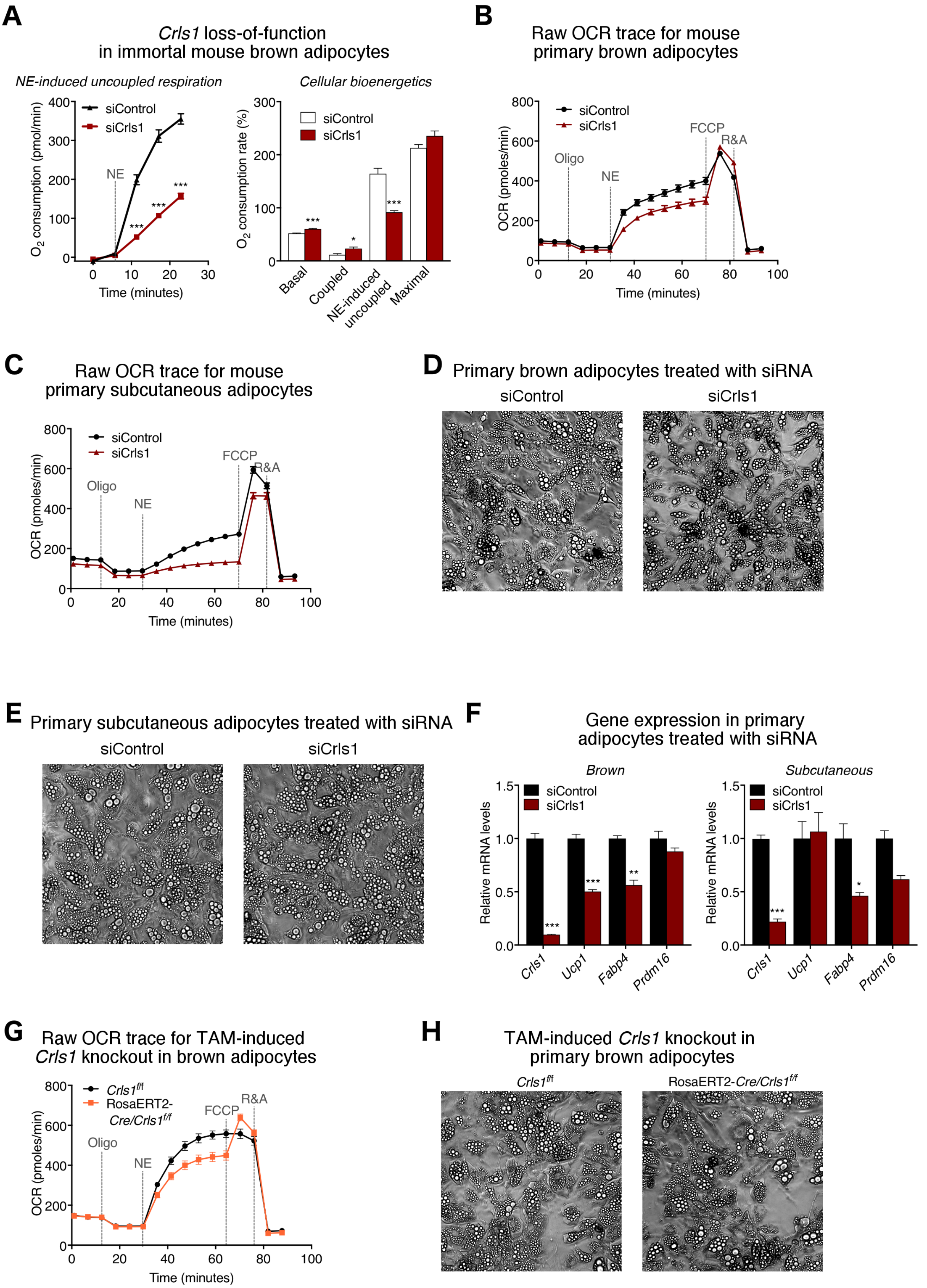
A
Crls1 loss-of-function
in immortal mouse brown adipocytes
NE-induced uncoupled respiration
O2 consumption (pmol/min)
siControl
siCrls1
NE
Time (minutes)
Cellular bioenergetics
O2 consumption rate (%)
Basal
Coupled
NE-induced uncoupled
Maximal
B
Raw OCR trace for mouse
primary brown adipocytes
OCR (pmoles/min)
Oligo
NE
FCCP
R&A
Time (minutes)
C
Raw OCR trace for mouse
primary subcutaneous adipocytes
D
Primary brown adipocytes treated with siRNA
siControl
siCrls1
E
Primary subcutaneous adipocytes treated with siRNA
siControl
siCrls1
F
Gene expression in primary
adipocytes treated with siRNA
Brown
Subcutaneous
Relative mRNA levels
Crls1
Ucp1
Fabp4
Prdm16
G
Raw OCR trace for TAM-induced
Crls1 knockout in brown adipocytes
Crls1f/f
RosaERT2-Cre/Crls1f/f
H
TAM-induced Crls1 knockout in
primary brown adipocytes
Crls1f/f
RosaERT2-Cre/Crls1f/f

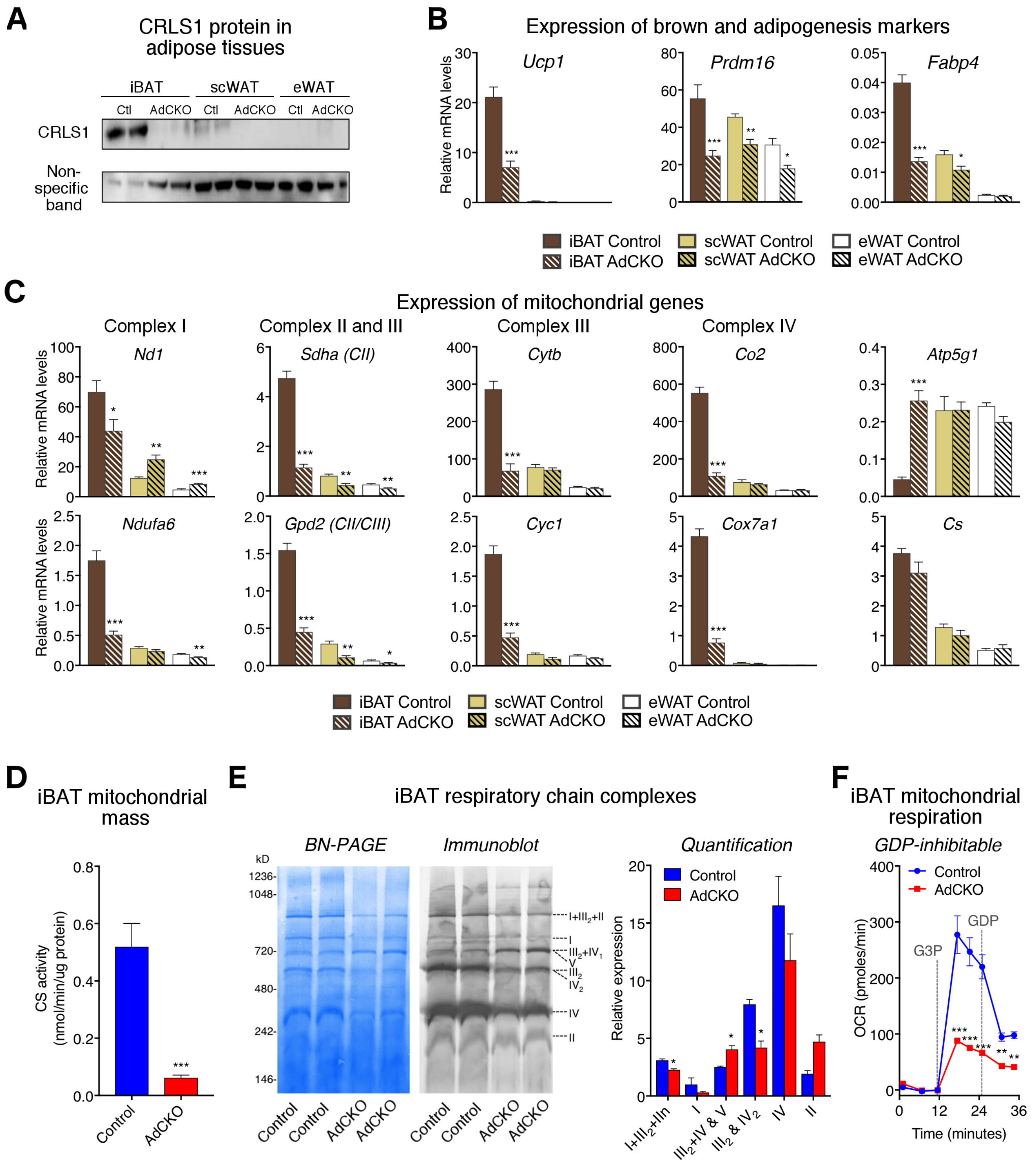
A
CRLS1 protein in adipose tissues
iBAT
scWAT
eWAT
Ctl
AdCKO
CRLS1
Non-specific band
B
Expression of brown and adipogenesis markers
Ucp1
Prdm16
Fabp4
Relative mRNA levels
iBAT Control
iBAT AdCKO
scWAT Control
scWAT AdCKO
eWAT Control
eWAT AdCKO
C
Expression of mitochondrial genes
Complex I
Complex II and III
Complex III
Complex IV
Nd1
Sdha (CII)
Cytb
Co2
Atp5g1
Ndufa6
Gpd2 (CII/CIII)
Cyc1
Cox7a1
Cs
D
iBAT mitochondrial mass
CS activity (nmol/min/ug protein)
Control
AdCKO
E
iBAT respiratory chain complexes
BN-PAGE
Immunoblot
Quantification
kD
Relative expression
F
iBAT mitochondrial respiration
GDP-inhibitable
OCR (pmoles/min)
G3P
GDP
Time (minutes)

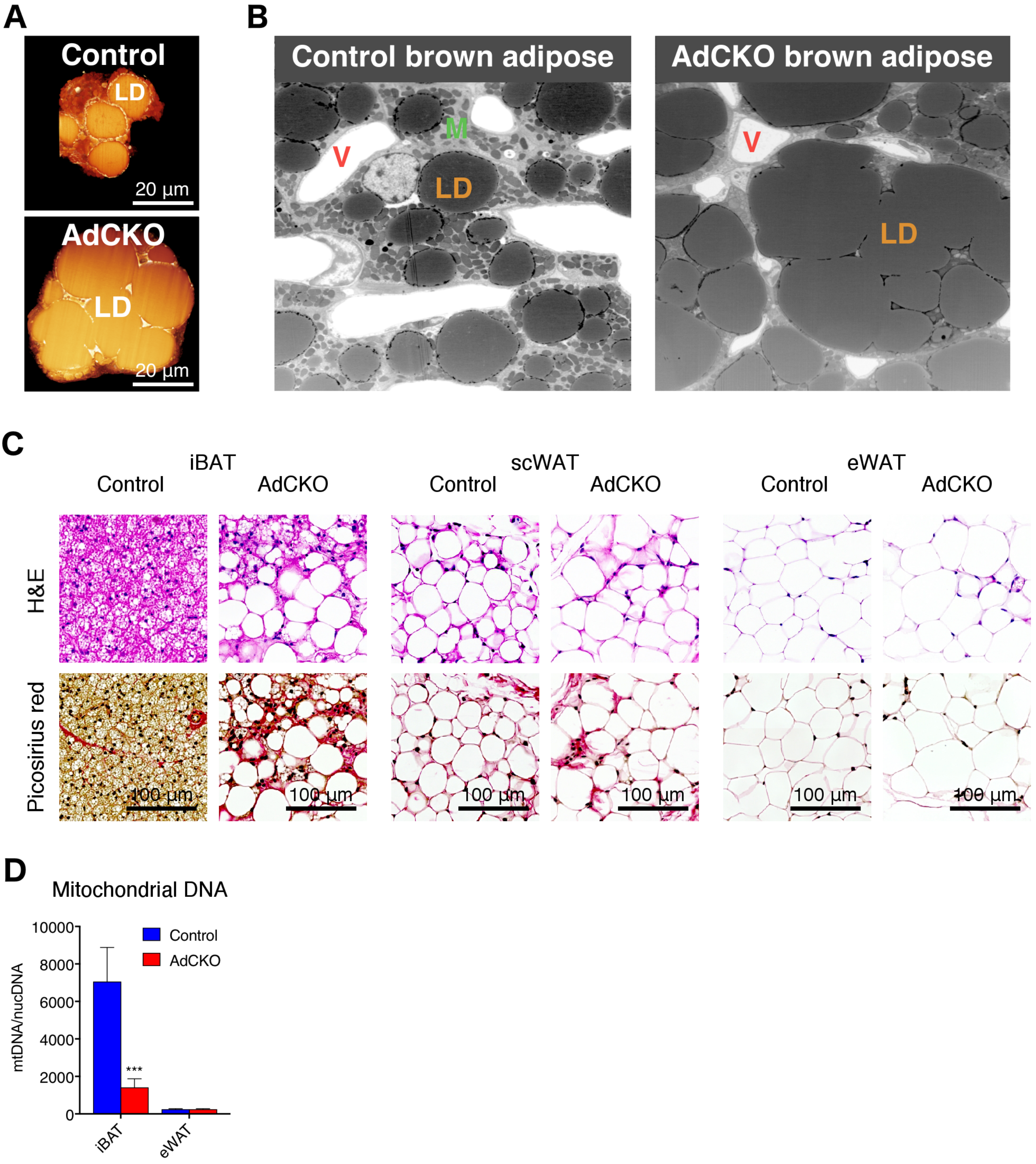

A
Control
LD
20 µm
AdCKO
LD
20 µm
B
Control brown adipose
V
M
LD
AdCKO brown adipose
V
LD
C
iBAT
Control
AdCKO
scWAT
Control
AdCKO
eWAT
Control
AdCKO
H&E
Picosirius red
100 µm
D
Mitochondrial DNA
mtDNA/nucDNA
10000
8000
6000
4000
2000
0
Control
AdCKO
***
iBAT
eWAT

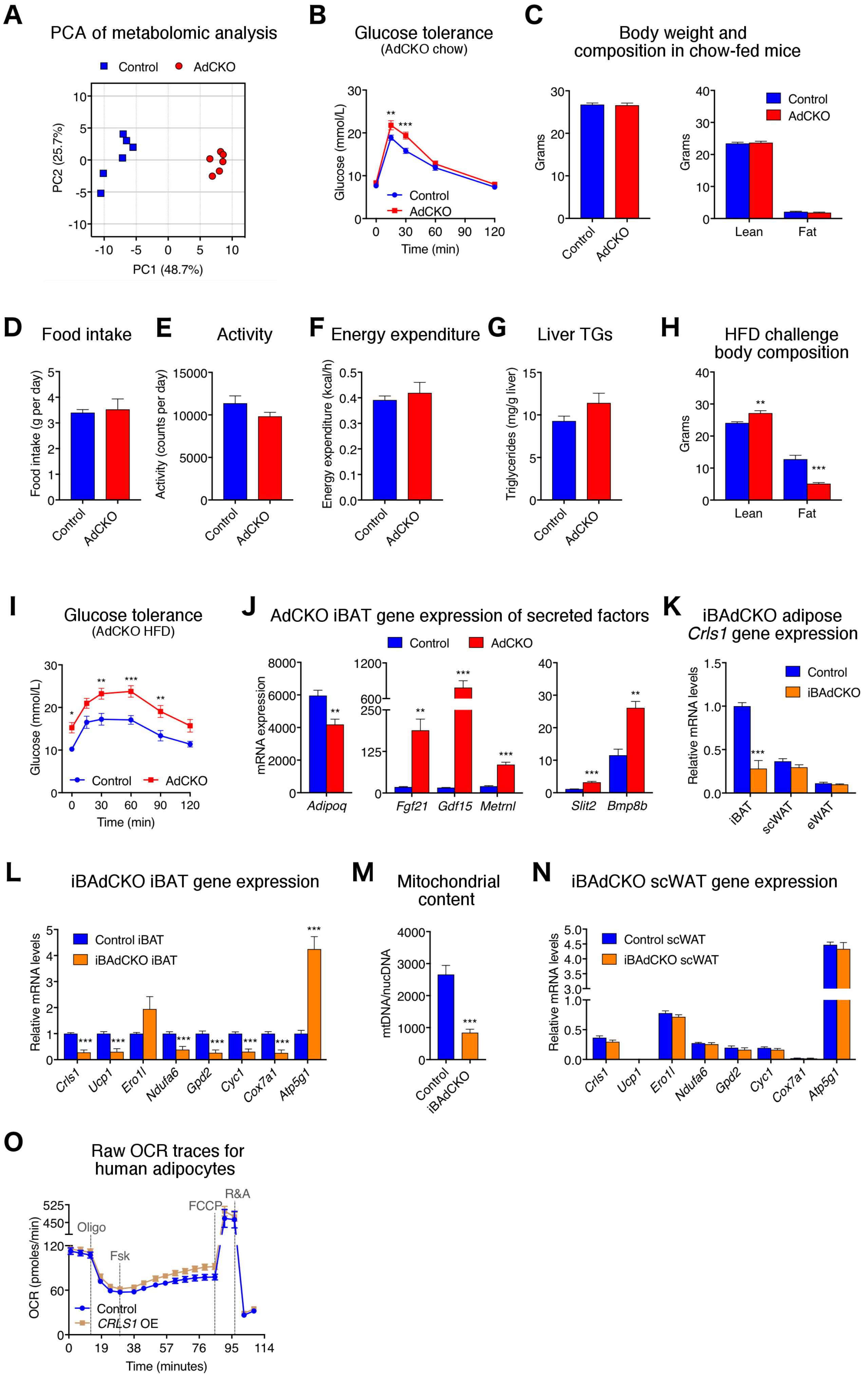
A
PCA of metabolomic analysis
Control
AdCKO
PC2 (25.7%)
PC1 (48.7%)
B
Glucose tolerance
(AdCKO chow)
Glucose (mmol/L)
Time (min)
C
Body weight and composition in chow-fed mice
Grams
Lean
Fat
D
Food intake
Food intake (g per day)
E
Activity
Activity (counts per day)
F
Energy expenditure
Energy expenditure (kcal/h)
G
Liver TGs
Triglycerides (mg/g liver)
H
HFD challenge body composition
I
Glucose tolerance
(AdCKO HFD)
J
AdCKO iBAT gene expression of secreted factors
mRNA expression
Adipoq
Fgf21
Gdf15
Metrnl
Slit2
Bmp8b
K
iBAdCKO adipose Crls1 gene expression
Relative mRNA levels
iBAdCKO
iBAT
scWAT
eWAT
L
iBAdCKO iBAT gene expression
Control iBAT
iBAdCKO iBAT
Crls1
Ucp1
Ero1l
Ndufa6
Gpd2
Cyc1
Cox7a1
Atp5g1
M
Mitochondrial content
mtDNA/nucDNA
N
iBAdCKO scWAT gene expression
Control scWAT
iBAdCKO scWAT
O
Raw OCR traces for human adipocytes
OCR (pmoles/min)
Oligo
Fsk
FCCP
R&A
CRLS1 OE
Time (minutes)

	CRLS1	
SNP	exm2254358	exm1524246
rs-id	rs149380663	rs41282950
Chromosome	20	20
Position (build 37)	6017765	6011934
Effect allele	C	T
Non effect allele	T	C
Minor Allele Frequency (MAF)	0.0005051	0.1291
Genotypes (HO/HE/WT)	0/16/15824	278/3509/11956
MAF in overlapping exome sequencing	0.0005089	0.1364
Annotation	coding-synonymous	missense
P (HWE)	1	0.3
**Fasting serum Insulin**	n=9072, b=0.78(0.3) , p=0.0099	n=9024, b=-0.02375(0.02209), p=0.2824
**2 h serum inuslin after OGTT**	n=6225, b=1.20(0.40), p=0.0023	n=6196, b=-0.000756(0.02611), p=0.9769
**HOMA-IR**	n=9066, b=0.78(0.3), p=0.0088	n=9018, b=-0.02109(0.022), p=0.3378
**BIGTT-SI**	n=5743, b=-1.20(0.39), p=0.0017	n=5715, b=0.0107(0.02634), p=0.6846
**Matsuda Insulin Senstivity Index**	n=5744, b=-1.20(0.4048), p=0.0031	n=5716, b=0.02111(0.02755), p=0.4436
**Body Mass Index**	n=14684, b=0.66(0.28), p=0.019	n=14596, b=0.002395(0.02011), p=0.9052
**Waist to hip Ratio**	n=12380, b=0.44(0.22), p=0.045	n=12309, b=-0.001856(0.01462), p=0.899

	PGS1				
SNP	exm1360386	exm-rs4129767	exm1903516	exm1360454	exm1360417
rs-id	rs2292642	rs4129767	rs0	rs0	rs145352765
Chromosome	17	17	17	17	17
Position (build 37)	76395430	76403984	76395468	76420046	76399916
Effect allele	C	C	A	C	G
Non effect allele	T	T	G	T	A
Minor Allele Frequency (MAF)	0.3941	0.492	0.0004419	0.0007261	0.00101
Genotypes (HO/HE/WT)	2481/7522/5835	3875/7834/4128	0/14/15826	0/23/15815	0/32/15808
MAF in overlapping exome sequencing	0.3908	NA	0.0005089	0.0007634	0.001018
Annotation	coding-synonymous-near-splice	intron	missense	missense	missense
P (HWE)	0.5	0.2	1	1	1
**Fasting serum Insulin**	n=9071, b=0.01473(0.01517), p=0.3316	n=9071, b=0.00756(0.01471), p=0.6073	n=9072, b=-0.2511(0.3317), p=0.449	n=9071, b=0.2003(0.2489), p=0.421	n=9072, b=-0.153(0.2414), p=0.5261
**2 h serum inuslin after OGTT**	n=6225, b=-0.02387(0.01821), p=0.1899	n=6225, b=-0.02502(0.01763), p=0.1559	n=6225, b=-0.6052(0.4924), p=0.2191	n=6225, b=0.1513(0.2844), p=0.5948	n=6225, b=-0.1597(0.2845), p=0.5745
**HOMA-IR**	n=9065, b=0.01591(0.01511), p=0.2923	n=9065, b=0.007687(0.01465), p=0.5997	n=9066, b=-0.3588(0.3302), p=0.2773	n=9065, b=0.1782(0.2478), p=0.472	n=9066, b=-0.1576(0.2403), p=0.5119
**BIGTT-SI**	n=5743, b=0.02049(0.0183), p=0.263	n=5743, b=0.03331(0.01772), p=0.06021	n=5743, b=0.8352(0.4737), p=0.07792	n=5743, b=-0.1838(0.2737), p=0.5018	n=5743, b=0.2029(0.2737), p=0.4585
**Matsuda Insulin Senstivity Index**	n=5744, b=0.01228(0.01914), p=0.5211	n=5744, b=0.02359(0.01854), p=0.2033	n=5744, b=0.241(0.4957), p=0.6269	n=5744, b=-0.1435(0.2864), p=0.6163	n=5744, b=0.1837(0.2864), p=0.5214
**Body Mass Index**	n=14682, b=-0.00141(0.01375), p=0.9183	n=14682, b=0.002514(0.0134), p=0.8512	n=14684, b=-0.4243(0.3206), p=0.1858	n=14682, b=0.1177(0.2465), p=0.6331	n=14684, b=0.8199(0.211), p=0.0001026
**Waist to hip Ratio**	n=12379, b=-0.02275(0.00999), p=0.0228	n=12379, b=-0.01782(0.009726), p=0.06701	n=12380, b=-0.1942(0.2324), p=0.4034	n=12378, b=0.2352(0.1724), p=0.1724	n=12380, b=0.06486(0.1512), p=0.668

	PTPMT1		
SNP	exm905472	exm905451	exm905478
rs-id	rs0	rs11537751	rs3207211
Chromosome	11	11	11
Position (build 37)	47593180	47587452	47594541
Effect allele	C	T	T
Non effect allele	G	C	C
Minor Allele Frequency (MAF)	0.004211	0.04476	0.0007891
Genotypes (HO/HE/WT)	0/133/15660	39/1340/14460	0/25/15815
MAF in overlapping exome sequencing	0.003053	0.04377	0.0005089
Annotation	stop-lost	missense	missense
P (HWE)	1	0.2	1
**Fasting serum Insulin**	n=9057, b=0.05328(0.1177), p=0.6508	n=9071, b=0.02202(0.0355), p=0.5351	n=9072, b=0.09986(0.2284), p=0.662
**2 h serum inuslin after OGTT**	n=6218, b=0.09233(0.1372), p=0.5011	n=6224, b=0.04286(0.04261), p=0.3145	n=6225, b=0.3108(0.3115), p=0.3185
**HOMA-IR**	n=9051, b=0.05272(0.1172), p=0.6528	n=9065, b=0.02628(0.03538), p=0.4577	n=9066, b=0.07631(0.2274), p=0.7372
**BIGTT-SI**	n=5737, b=-0.1006(0.1361), p=0.4596	n=5742, b=-0.05365(0.0426), p=0.2079	n=5743, b=-0.06158(0.2998), p=0.8372
**Matsuda Insulin Senstivity Index**	n=5738, b=-0.03154(0.1424), p=0.8247	n=5743, b=-0.05512(0.04457), p=0.2162	n=5744, b=-0.07592(0.3137), p=0.8088
**Body Mass Index**	n=14642, b=0.06366(0.1038), p=0.5398	n=14683, b=0.03041(0.03242), p=0.3483	n=14684, b=0.03309(0.2313), p=0.8862
**Waist to hip Ratio**	n=12339, b=-0.05923(0.07553), p=0.433	n=12379, b=0.007127(0.02335), p=0.7602	n=12380, b=0.02085(0.1682), p=0.9014

Species	Gene	Forward (Sequence 5'-3')	Reverse (Sequence 5'-3')	Use
human	*36B4*	TTTGTGTTCACCAAGGAGGA	GTGACTTCACATGGGGCAAT	mRNA quantification
human	*CRLS1*	GCTTGGCCCCAGTTCTGG	GGCCCAGTTTCGAGCAATAA	mRNA quantification
human	*FABP4*	TACTGGGCCAGGAATTTGAC	TACCAGGACACCCCCATCTA	mRNA quantification
human	*PRDM16*	CGGCAAAGGAGACAGACTTC	CATCCACGCAGAACTTCTCA	mRNA quantification
Human	*UCP1*	AAGGCTTGACGGGTCTTTG	CGATAAGAGCCGACACCAAG	mRNA quantification
mouse	*36b4*	TCATCCAGCAGGTGTTTGACA	GGCACCGAGGCAACAGTT	mRNA quantification
mouse	*Atp5g1*	TTGGCACAGTGTTTGGTAGC	CAAACCCCAGAATGGCATAG	mRNA quantification
mouse	*Co2*	ATTTAGTCGGCCTGGGATG	ACCGAGTCGTTCTGCCAATA	mRNA and mitochondrial DNA quantification
mouse	*Cox7a1*	AAAACCGTGTGGCAGAGAAG	CAGCGTCATGGTCAGTCTGT	mRNA quantification
mouse	*Crls1*	GGGCTACCTGATTCTTGAAGA	GGCCCAGTTTCGAGCAATAA	mRNA quantification
mouse	*Cs*	GGGGTGCTGCTCCAGTACTATG	AAAGGCCCCTGAAACAAAACAAAA	mRNA quantification
mouse	*Cyc1*	GAGCTTTACCCCCTGACCTC	GTAGCCAGTGAGCAGGGAAA	mRNA quantification
mouse	*Cytb*	ATTCCTTCATGTCGGACGAG	CTGTGGCTATGACTGCGAAC	mRNA and mitochondrial DNA quantification
mouse	*Ddit3 (Chop-10)*	TATCTCATCCCCAGGAAACG	GATGTGCGTGTGACCTCTGT	mRNA quantification
mouse	*Ero1l*	CTTCAGTGGACCAAGCATGA	GCCCCTTGTAGCCTGTGTAG	mRNA quantification
mouse	*Fabp4*	GGGCGTGGAATTCGATGAAA	GGTCGACTTTCCATCCCACT	mRNA quantification
mouse	*Gpd2*	AAAACGTTGTTCCCATCTGC	CACTGGTTGCAGGATCAAGA	mRNA quantification
mouse	*Nd1*	GGATCCGAGCATCTTATCCA	GGTGGTACTCCCGCTGTAAA	mRNA and mitochondrial DNA quantification
mouse	*Nd2*	AACCCACGATCAACTGAAGC	GCGAGGCCTAGTTTTATGGA	mRNA and mitochondrial DNA quantification
mouse	*Ndufa6*	ACCCCAGAGTGGTTGATCTG	GGAAAAACCGCATAACGTGT	mRNA quantification
mouse	*Nuclear DNA-Pparg*	TTTGGAATTCTCACAAAACTTCA	TTTTCTACTGCTGGGGATGG	mitochondrial DNA quantification
mouse	*Prdm16*	CCTGTGGGAGTCCTGAAAGA	CAGCTTCTCCGTCATGGTTT	mRNA quantification
mouse	*Sdha*	AACACTGGAGGAAGCACACC	GCACAGTCAGCCTCATTCAA	mRNA quantification
mouse	*Ucp1*	GGATTGGCCTCTACGACTCA	TAAGCCGGCTGAGATCTTGT	mRNA quantification
mouse	*Nuclear DNA-Ucp1*	GAGGCAGTCAAGAGCAGCTT	GCCCAATACACAAGCCCTAA	mitochondrial DNA quantification

SUPPLEMENTAL FIGURE LEGENDS

Figure S1. Proteomic analysis of iBAT from cold exposed mice. Related to Figure 1. (**A**) Principle component analysis of iBAT proteomics data. (**B**) iBAT protein levels of known markers of thermogenesis from proteomic analysis presented in Figure 1A; Glycerokinase (GK), Elongation Of Very Long Chain Fatty Acids 3 (ELOVL3), Glycerol-3-phosphate dehydrogenase 1 (GPD1), Acyl-CoA Synthetase Long-Chain Family Member 5 (ACSL5), and Acetyl-CoA Carboxylase Alpha (ACACA) (n=4 for 29°C and 5°C 3 weeks, n=3 for other groups; one-way ANOVA). AU=arbitrary units. Data are presented as means ± SEM. *$p < 0.05$; **$p < 0.01$; ***$p < 0.001$.

Figure S2. Lipidomic analysis of iBAT and scWAT from cold exposed mice. Related to Figure 2. (**A**) Heatmap of Z score transformed data for 250 significantly altered lipids from targeted lipidomic analysis of interscapular BAT from mice housed at thermoneutrality or exposed to 5°C cold for 3 hours, 3 days, or 3 weeks (n=4 per group, ANOVA). (**B**) Heatmap of Z score transformed data for the 32 significantly altered lipids from targeted lipidomic analysis of subcutaneous WAT from mice housed at thermoneutrality or exposed to 5°C cold for 3 weeks (n=4 per group, *t* test). (**C**) Heatmaps of $\log_2$ (fold change cold treated/thermoneutrality) for the 14 phosphatidylglycerol (PG) species measured by targeted lipidomics of iBAT and scWAT from mice housed at thermoneutrality or exposed to 5°C cold for up to 3 weeks (n=4 per group, one-way ANOVA for iBAT and *t* tests for scWAT). Normalized lipid quantities (pmol lipid/mg protein) and donut charts of PG (**D**) and CL (**E**) species from targeted lipidomic analysis of interscapular BAT and subcutaneous WAT from mice housed at thermoneutrality or exposed to 5°C cold for 3 weeks (n=4 per group; lipid species increased by cold are shades of maroon and those decreased are shades of dark blue). Data are presented as means ± SEM. *$p < 0.05$; **$p < 0.01$; ***$p < 0.001$.

Figure S3. Respirometry of adipocytes with *Crls1* loss of function. Related to Figure 3. (**A**) Oxygen consumption profiles from control and *Crls1* siRNA-treated immortalized brown adipocytes following addition of oligomycin and NE stimulation (RM two-way ANOVA). Quantified levels of basal mitochondrial, ATP synthesis-coupled, NE-induced uncoupled, and maximal respiration are provided to the right (*t* tests). Raw OCR traces for cellular respirometry on siRNA-treated primary (**B**) brown and (**C**) subcutaneous adipocytes. (**D**) Representative images (200X magnification) of primary brown adipocytes treated with siRNA. (**E**) Representative images (200X magnification) of primary subcutaneous adipocytes treated with siRNA. (**F**) mRNA levels of *Crls1* and thermogenic (*Ucp1* and *Prdm16*) and adipogenic (*Fabp4*) markers in siRNA treated primary adipocytes. (**G**) Raw OCR traces for cellular respirometry on primary brown adipocytes with TAM-induced *Crls1* knockout. (**H**) Representative images (200X magnification) of primary brown adipocytes from Rosa26ERT2-*Cre*/*Crls1*$^{f/f}$ mice with TAM-induced *Crls1* knockout. Data are presented as means ± SEM. *$p < 0.05$; **$p < 0.01$; ***$p < 0.001$.

Figure S4. Analysis of mitochondrial genes and proteins in AdCKO adipose tissue. Related to Figure 4. (**A**) Western blot of CRLS1 in adipose tissues (iBAT, scWAT and eWAT) from AdCKO mice. (**B**) mRNA levels of thermogenic (*Ucp1* and *Prdm16*) and adipogenic (*Fabp4*) markers in control and AdCKO adipose tissues (n=6-7, two-way ANOVA). (**C**) mRNA levels of genes for the electron transport chain complexes I-IV (*Nd1*, *Ndufa6*, *Sdha*, *Gpd2*, *Cytb*, *Cyc1*, *Co2* and *Cox7a1*), as well as *Atp5g1* and *Cs* in control and AdCKO adipose tissues (20 week old males; n=6-7 per group). (**D**) Mitochondrial mass (as measured by citrate synthase (CS) activity from control and AdCKO iBAT (n=4-7 per group, *t* test). (**E**) Respiratory chain complexes and supercomplexes resolved by blue native (BN) page and immunoblot of isolated mitochondrial protein from control and AdCKO iBAT. Quantification is from the immunoblot (*t* tests). (**F**) Respirometry of mitochondria isolated from AdCKO iBAT and given G3P substrate, followed

by GDP to inhibit UCP1 activity. Data are presented as means ± SEM. *$p < 0.05$; **$p < 0.01$; ***$p < 0.001$.

Figure S5. Electron microscopy and histology of AdCKO adipose tissue. Related to Figure 4. (**A**) Representative cross-sections showing lipid droplets of control and AdCKO adipocytes derived from 3-dimensional reconstructions of AdCKO adipocytes generated from Dual-beam FIB SEM stacks. Representative SEM micrographs of (**B**) control and AdCKO (**C**) brown adipose tissue at 5kX magnification generated during Dual-beam FIB SEM. (**D**) Representative H&E and Picosirius red stained histology sections of iBAT, scWAT and eWAT from control and AdCKO mice. (**E**) Mitochondrial DNA (mtDNA) content of control and AdCKO brown (iBAT) and epididymal white (eWAT) adipose tissues (n=5 per group, *t* tests) measured as a ratio of mtDNA to nuclear DNA (nucDNA). Data are presented as means ± SEM. ***$p < 0.001$.

Figure S6. Metabolic phenotyping and gene expression analysis of AdCKO and iBAdCKO adipose tissue. Related to Figures 6 and 7. (**A**) Principle component analysis of metabolomic data from iBAT of control and AdCKO mice (n=6 per group). (**B**) Glucose tolerance of control and AdCKO mice on chow diet (n=11 per group, two-way ANOVA). (**C**) Body weight and composition of control (n=15) and AdCKO (n=13) mice on chow diet; (two-way ANOVA). (**D**) Daily food intake, (**E**) activity counts, and (**F**) energy expenditure of control and AdCKO mice (n=6 per group). (**G**) Hepatic triglyceride concentrations in chow fed control and AdCKO mice (n=7 per group). (**H**) Body weight and composition (n=11 per group) of control and AdCKO mice on HFD (n=6-15 per group; *t* tests). (**I**) Glucose tolerance of control and AdCKO mice on HFD (control n=10, AdCKO n=9; two-way ANOVA). (**J**) Gene expression of secreted factors from RNA-seq of AdCKO iBAT. (**K**) *Crls1* gene expression in iBAdCKO adipose tissues and (**L**) gene expression in iBAdCKO iBAT (n=5, *t* tests). (**M**) Mitochondrial DNA (mtDNA) content of control and iBAdCKO iBAT (n=8, *t* test) measured as a ratio of mtDNA to nuclear DNA

(nucDNA). (**N**) Gene expression in iBAdCKO scWAT (n=5, *t* tests). (**O**) Raw OCR traces for cellular respirometry on human adipocytes with CRISPRa-SAM mediated expression of *CRLS1*. Data are presented as means ± SEM. $*p < 0.05$; $**p < 0.01$; $***p < 0.001$.

SUPPLEMENTAL TABLES

Table S1. Table of human genetic association data for variants in CL synthesis and remodeling genes. Related to Figure 7.

Table S2. Sequences of primers used for gene expression analysis and mitochondrial DNA quantification. Related to STAR Methods.
